# Influence of Fluorination on the Conformational Properties and Hydrogen-Bond Acidity of Benzyl Alcohol Derivatives

**DOI:** 10.1002/chem.201501171

**Published:** 2015-06-30

**Authors:** Elena Bogdan, Guillaume Compain, Lewis Mtashobya, Jean-Yves Le Questel, François Besseau, Nicolas Galland, Bruno Linclau, Jérôme Graton

**Affiliations:** [a] CEISAM UMR CNRS 6230, Faculté des Sciences et des Techniques, Université de Nantes 2 rue de la Houssinière, BP 92208, 44322 NANTES Cedex 3 (France), Fax: (+3) 2-51-12-54-02 E-mail: jerome.graton@univ-nantes.fr; [b] Department of Chemistry, University of Southampton Highfield, Southampton SO17 1BJ (UK), Fax: (+44) 23-8059-6805 E-mail: bruno.linclau@soton.ac.uk

**Keywords:** hydrogen-bond acidity, benzylic alcohols, conformation analysis, fluorine

## Abstract

The effect of fluorination on the conformational and hydrogen-bond (HB)-donating properties of a series of benzyl alcohols has been investigated experimentally by IR spectroscopy and theoretically with quantum chemical methods (ab initio (MP2) and DFT (MPWB1K)). It was found that *o*-fluorination generally resulted in an increase in the HB acidity of the hydroxyl group, whereas a decrease was observed upon *o*,*o′*-difluorination. Computational analysis showed that the conformational landscapes of the title compounds are strongly influenced by the presence of *o*-fluorine atoms. Intramolecular interaction descriptors based on AIM, NCI and NBO analyses reveal that, in addition to an intramolecular OH⋅⋅⋅F interaction, secondary CH⋅⋅⋅F and/or CH⋅⋅⋅O interactions also occur, contributing to the stabilisation of the various conformations, and influencing the overall HB properties of the alcohol group. The benzyl alcohol HB-donating capacity trends are properly described by an electrostatic potential based descriptor calculated at the MPWB1K/6-31+G(d,p) level of theory, provided solvation effects are taken into account for these flexible HB donors.

## Introduction

The fluorination of organic compounds to modify their properties is having a major impact in many chemistry-related fields such as medicinal chemistry,[1] agrochemistry,[2] materials science[3] and crystal engineering.[4] The high fluorine electronegativity, with the resulting highly polarised C–F bond and nonpolarisable fluorine lone pairs, is at the origin of a multitude of effects resulting from the introduction of one or more fluorine atoms.[5] Fundamental studies aimed at improving our understanding of the effects of fluorination in organic compounds are still ongoing. Significant and sometimes unexpected consequences of fluorination on the physical and chemical properties of adjacent functional groups[6] or regarding C–F mediated inter- and intramolecular interactions, continue to be described.[7] Organofluorine chemists are especially captivated by the ability of fluorine to behave as a hydrogen-bond (HB) acceptor,[8] and it is now accepted, through key contributions from Vulpetti and Dalvit[7c] as well as Laurence and co-workers,[9] that organofluorine can act as a weak HB acceptor. Furthermore, seminal works by Vasella, Bernet and Gouverneur have highlighted OH⋅⋅⋅F intramolecular hydrogen bonds (IMHBs) by using NMR techniques.[10]

Recently, we have experimentally determined HB-donating capacities (or HB acidities) of fluorohydrins through the adaptation of an established[11] procedure by using FTIR spectroscopy.[6c] The insights revealed in this study, for example the influence of OH⋅⋅⋅F IMHB interactions on alcohol hydrogen-bond properties, pointed out the need for comprehensive investigations on a wide range of fluorinated compounds to probe the effects of fluorine on HB interactions in diverse chemical environments, and to optimise HB property prediction tools.

Herein we report on the influence of *ortho*-fluorination on the hydrogen-bond-donating capacity of benzyl alcohols through a combined experimental and theoretical approach. The experimental HB acidities (p*K*_AHY_) are presented and rationalised by quantum chemistry calculations, including detailed conformational analysis, to allow insights to be gained on the influence of the fluorine atom(s) on the conformational features of substituted benzyl alcohols. Atoms In Molecules (AIM),[12] Noncovalent Interaction (NCI)[13] and Natural Bond Orbital (NBO)[14] analyses have been performed to provide an accurate description of the different IMHB interactions occurring in the various compounds. In the final part of this work, we show the feasibility of accurately predicting the HB acidity values of the substrates involved by using an electrostatic-based descriptor (*V*_α_(*r*))[15] computed for the various molecules.

Benzyl alcohols are common building blocks of drugs (e.g., antimuscarinic drugs (fesoterodine), neuroprotective agents, anticonvulsant agents (gastrodin)), and their conformational preferences are still a matter of debate.[16]

**Figure 1:**
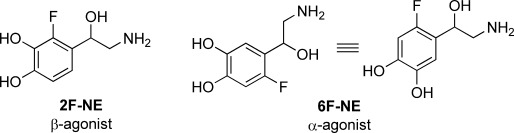
Fluorinated norepinephrine analogues with different agonist activities.

An interesting effect of fluorine substitution has been reported for ring-hydroxylated biogenic amines such as norepinephrine (Figure 1). Depending on the position of the fluorine, the analogues were shown to have markedly different agonist properties.[17] Intramolecular hydrogen-bonding effects and/or dipole-dipole repulsions between the COH and CF moieties have been considered as factors that could result in conformational preferences that are favourable for binding to α- or β-adrenergic receptors.[17–18] A more recent explanation involves preferential orientation of the C–F bond of both 2F-NE and 6F-NE to an asparagine residue, resulting in a different presentation of the aromatic alcohol groups.[19]

## Results and Discussion

### Synthesis

**Figure 2:**
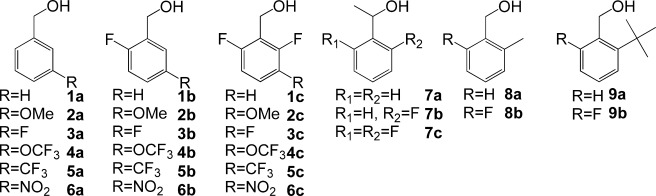
Chemical structures of the benzyl alcohol derivatives under study.

The synthesis of substrates **1 b**, **2 b**, **6 b** and **6 c** (Figure 2) is detailed in the Supporting Information. All other compounds were purchased. Compounds **4 c**, **5 c**, **9 a** and **9 b** were only investigated computationally.

### Conformational IR Analysis of the Hydroxyl Stretching Region

**Figure 3:**
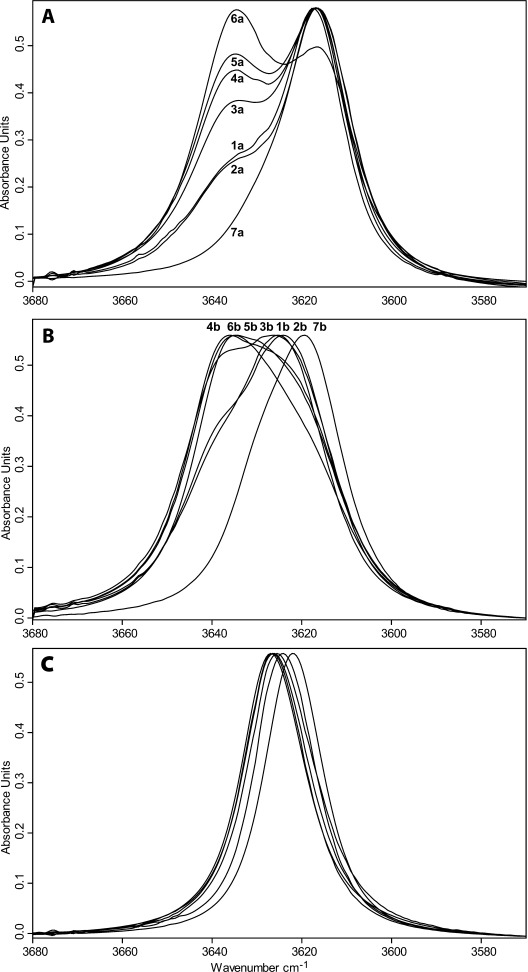
IR spectra in the *ν*_OH_ stretching region of A) benzyl alcohols, B) 2-fluorobenzyl alcohols and C) 2,6-difluorobenzyl alcohols.

The experimental data set investigated in this study is composed of eight reference benzyl alcohols **1 a**–**8 a**, eight monofluorinated 2-fluorobenzyl alcohols **1 b**–**8 b** and five difluorinated 2,6-difluorobenzyl alcohol derivatives **1 c**–**3 c**, **6 c** and **7 c**. The *ν*_OH_ bands of the title compounds at 25 °C in dilute CCl_4_ solutions are shown in Figure 3.

For most of the nonfluorinated benzyl alcohols, the *ν*_OH_ region is rather complex, with two bands separated by ca. 20 cm^−1^, which is indicative of different conformations. A deconvolution of the absorption spectra in this region has therefore been carried out; the resulting *ν*_OH_ stretching frequencies are reported in Table 1. For series **a**, without any *ortho* substituent, the low-frequency band *ν*_OH(2)_ peaked at 3616 cm^−1^ irrespective of the nature of the *meta* substituent. The position of the *ν*_OH(1)_ absorption band is measured at a slightly higher frequency, 3629 cm^−1^ for **1 a** and **2 a** , and 3635 cm^−1^ for **3 a**–**6 a** (Figure 3 A, Table 1). However, their relative intensity is dependent on the substitution: whereas *ν*_OH(2)_ is shown to be the main band, the contribution of *ν*_OH(1)_ increases with increasing electron-withdrawing effect of the *meta* substituent. With the nitro-derivative **6 a**, the band at 3635 cm^−1^ shows the largest contribution. Only one conformer absorbing at low frequency (3617 cm^−1^) is observed in the presence of a methyl group in the α-position (**7 a**), whereas when the methyl group is in the *o*-position (**8 a**) (not shown), the band profile is similar to that of **1 a**.

**Table 1:** Experimental spectroscopic features, *ν*_OH_, ε_OH_ and Δ*ν*_OH_, and HB acidity properties, p*K*_AHY_ and Δ*G*_AHY_, of benzyl alcohols under study

Entry	*ν*_OH(1)_ [cm^−1^]	*ν*_OH(2)_ [cm^−1^]	ε_OH_ [L mol^−1^ cm^−1^]	p*K*_AHY_	Δ*G*_AHY_ [kJ mol^−1^]	Δ*ν*_OH_ [cm^−1^]^[a]^
**1 a**	3629	3616	73	1.03	−5.9	193
**1 b**	3639	3624	75	1.16	−6.6	207
**1 c**	–^[b]^	3627	117	0.94	−5.4	222
**2 a**	3629	3616	76	1.06	−6.1	197
**2 b**	3639	3623	74	1.21	−6.9	208
**2 c**	–^[b]^	3627	108	0.86	−4.9	228
**3 a**	3635	3616	72	1.32	−7.5	205
**3 b**	3638	3623	66	1.48	−8.4	224
**3 c**	–^[b]^	3626	107	1.21	−6.9	229
**4 a**	3635	3616	70	1.46	−8.3	231
**4 b**	3638	3622	78	1.70	−9.7	241
**5 a**	3635	3616	70	1.48	−8.4	213
**5 b**	3638	3622	77	1.67	−9.5	240
**6 a**	3635	3616	73	1.79	−10.2	245
**6 b**	3636	3621	88	1.98	−11.3	259
**6 c**	–^[b]^	3626	123	1.69	−9.6	276
**7 a**	–^[b]^	3617	86	0.96	−5.5	193
**7 b**	3630	3619	80	1.05	−6.0	201
**7 c**	–^[b]^	3622	115	0.70	−4.0	206
**8 a**	3637	3619	73	0.99	−5.7	197
**8 b**	–^[b]^	3624	110	0.94	−5.4	215

[a] Calculated from the *ν*_OH(2)_ value. [b] Not observed.

Within series **b** (Figure 3 B), the *ν*_OH_ bands are slightly blueshifted by ca. 3–10 cm^−1^, and are less resolved. The *ν*_OH(1)_ contribution appears to be higher than in series **a**.

With the 2,6-difluoro series **c** (Figure 3 C), only one stretching *ν*_OH_ band is observed and its half-width is significantly smaller than in compounds **a** and **b**. This feature might indicate that the conformational flexibility around the hydroxyl moiety is lost in these derivatives, which would reduce the number of existing conformers with respect to series **a** and **b**. Similarly, compound **8 b**, having two *o*-substituents, exhibits a *ν*_OH_ stretching band with a small half-width (not shown), closer to the profile of compounds of series **c** than to the series **b**.

### Computational Analysis

#### Introduction: Conformational Studies

The conformational properties of benzyl alcohol, described either through experimental or theoretical studies, are still a matter of debate, whereas they seem to be more established for benzylic compounds, C_6_H_5_CH_2_X. When X is an alkyl group or a halide, the C-C-C-X dihedral angle was shown to be 90°, with the C–X bond in a plane orthogonal to the benzene ring.[20] This *perpendicular* conformation minimises steric repulsive effects between the -CH_2_X group and the phenyl ring. If the X group contains a triple bond (ethynyl or cyanide), the dihedral angle is near 0°, with the C≡C bond lying in the plane of the phenyl ring.[21] The preference for this *planar* structure has been rationalised by the presence of a stabilising CH⋅⋅⋅π HB interaction between an aromatic CH bond and the triple bond π-electron cloud.

**Figure 4:**
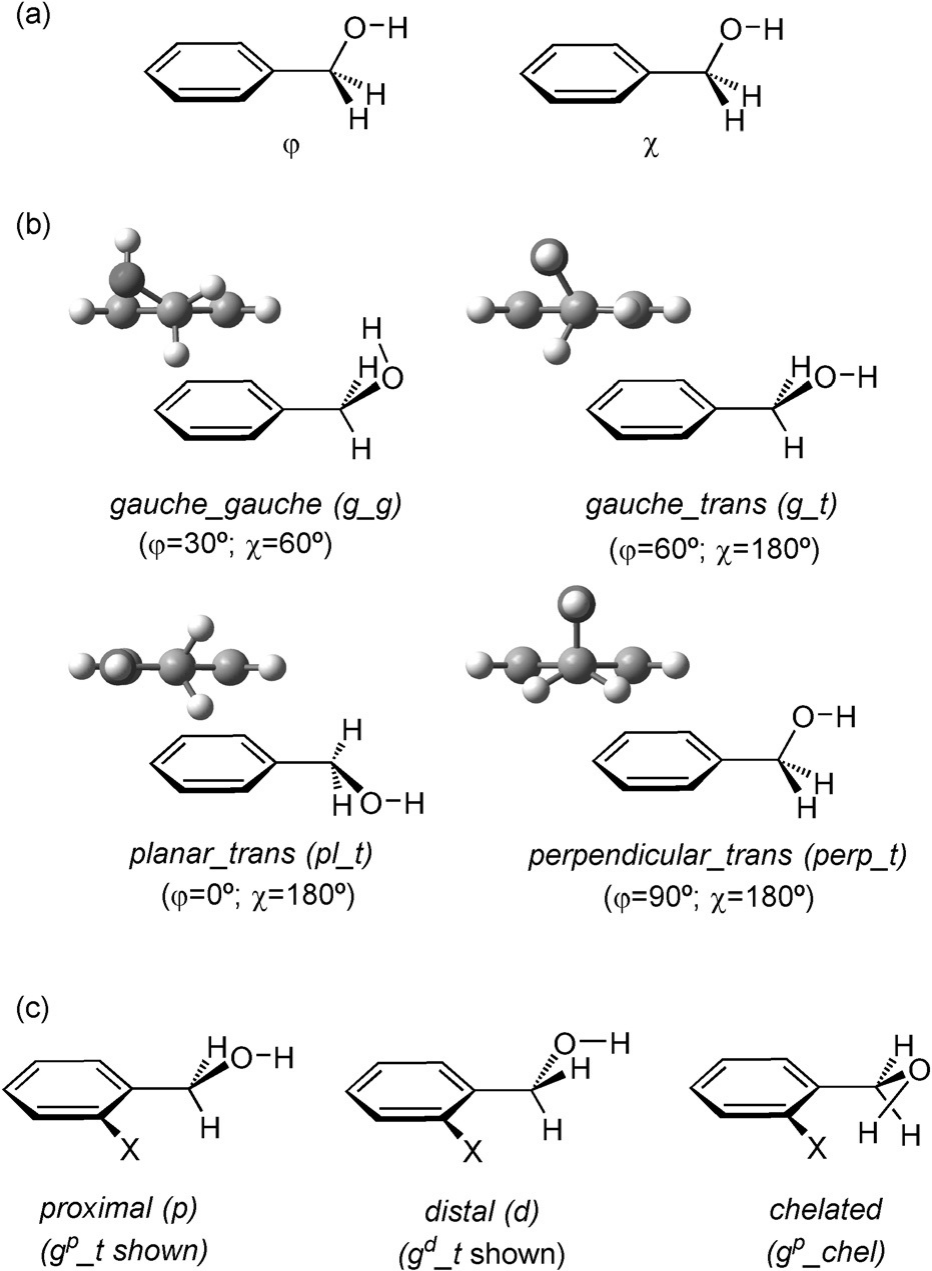
Benzyl alcohol conformations: a) Description of the *ϕ* and *χ* dihedral angles of interest. b) Main conformations encountered in the benzyl alcohol structures in Newman representation along the C_*ipso*_–C_α_ bond. c) Definition of *proximal*, *distal* and *chelated gauche* conformations for substituted benzyl alcohols. See the Supporting Information for detailed aspects of the nomenclature used.

Although benzyl alcohol has been the subject of extensive experimental and theoretical studies,[16a, 22] there remains a degree of uncertainty about the number of stable conformers and their relative stabilities. The hydroxymethyl side chain is flexible and it is generally accepted that several conformers simultaneously exist in the gas phase, a situation similar to benzylamine and its derivatives.[23] The main suggested conformers of benzyl alcohol derivatives, defined by the ϕ (C_*ortho*_C_*ipso*_C_α_O) and χ (C_*ipso*_C_α_OH) dihedral angles, are the *gauche*_*gauche* (*g*_*g*, also sometimes referred to as *gauche*_*cis*), *gauche*_*trans* (*g*_*t*), *planar* (*pl*) and *perpendicular* (*perp*) conformers (Figure 4 a,b and the Supporting Information for further details). In monofluorinated benzyl alcohols, the introduction of a methyl group at the C_α_ carbon was found to have a significant influence on the preferred conformation compared with *o*-fluoro benzyl alcohol, preferentially showing an OH⋅⋅⋅F IMHB,[24] to 2-fluoro-α-methylbenzyl alcohol,[25] mainly stabilised with an OH⋅⋅⋅π interaction. Theoretical calculations in the gas phase generally attribute the absolute minimum to the *gauche*_*gauche* conformation.[16a, b, d] However, depending on the theoretical methods and experimental techniques selected, different geometries have been proposed for the first local minimum,[16a, 22] and the situation is even more challenging in the solution state. In a previous study based on IR spectroscopy in CCl_4_ and CS_2_ solutions, the presence of *gauche* and *planar* conformations was suggested, but the *perpendicular* conformation could not be excluded.[22a]

Hence, to support our experimental HB measurements, a detailed conformational analysis of the benzyl alcohol derivatives was required. Following the recommendations of Basso,[26] our analysis involved a polarisable continuum model (PCM) explicitly describing the hydrogen atoms to take into account the solvation effects on the conformational equilibrium of benzyl alcohol derivatives at the MP2/6-311++G(2d,p) level of theory, after optimisation at the IEFPCM-MPWB1K/6-31+G(d,p) level. The results are given in Tables 2–4, wherein the theoretical frequencies of the *ν*_OH_ stretching vibrations are also listed, and details of the computed relative free energies and conformer populations are provided in the Supporting Information (Table S1).

**Table 2:** Calculated populations *p*_i_ of the conformers of nonfluorinated benzyl alcohol derivatives (1 a–9 a) in CCl_4_ medium at the IEFPCM-MP2/6-311++G(2d,p) level of theory. Optimised dihedral angles *ϕ* (C_*ortho*_C_*ipso*_C_α_O) and *χ* (C_*ipso*_C_α_OH) characterising the hydroxyl moiety orientation and calculated *ν*_OH_ stretching frequencies (IEFPCM-MPWB1K/6-31+G(d,p))^[a]^

Compound	Conformer	*p*_i_ [%]	ϕ [°]	χ [°]	*ν*_OH_ [cm^−1^]^[b]^
**1 a**	*g*_*g*	82	33	60	3616
	*pl*	18	0	180	3646
**2 a**	*g*_*g*	61	26/36	59/61	3616
	*g*_*t*	21	10/15	173/174	3645
	*pl*	18	0	180	3645
**3 a**	*g*_*g*	57	28/29	63/64	3618
	*pl*	43	0/5	177/180	3646
**4 a**	*g*_*g*	82	26/31	64/66	3618
	*pl*	18	0/8	175/180	3645
**5 a**	*g*_*g*	74	21	65/67	3618
	*pl*	26	0/2	179/180	3645
**6 a**	*g*_*g*	68	23/30	68/69	3619
	*pl*	32	0	180	3645
**7 a**	*g*_*g*	92	36/39	57/61	3609
	*g*_*t*	8	19	174	3630
**8 a**	*g*_*g*	78	10/69	55/66	3619
	*pl*	14	0	180	3646
	*g*_*t*	8	66	174	3626
**9 a**	*g*_*g*	89	61/87	47/58	3615
	*perp*	8	80	173	3618
	*g*_*t*	3	22/63	174/176	3640/3623

[a] See Figure 4 for conformer definitions. When relevant, *proximal*/*distal* conformations are grouped together. The detailed computed relative free energies and conformer populations are provided in the Supporting Information (Table S1). [b] Scaled by 3616/3972=0.91, the ratio between the calculated and the experimental *ν*_OH_ value for benzylalcohol *g*_*g* conformer.

**Table 3:** Calculated populations *p*_i_ of the conformers of monofluorinated benzyl alcohol derivatives (1 b–9 b) in CCl_4_ medium at the IEFPCM-MP2/6-311++G(2d,p) level of theory. Optimised dihedral angles ϕ (C_ortho_C_ipso_C_α_O) and χ (C_ipso_C_α_OH) characterising the hydroxyl moiety orientation and *ν*_OH_ stretching frequencies (IEFPCM-MPWB1K/6-31+G(d,p)^[a]^

Compound	Conformer	*p*_i_ [%]	ϕ [°]	χ [°]	*ν*_OH_ [cm^−1^]^[b]^
**1 b**	*g*^*p*^_*g*	29	69	57	3621
	*g*^*p*^_*chel*	21	61	67	3626
	*g*^*d*^_*g*	20	18	69	3620
	*pl*^*d*^	17	0	180	3647
	*perp*_*t*	13	82	171	3627
**2 b**	*g*^*d*^_*g*	31	14/21	69	3620
	*g*^*p*^_*chel*	24	60	65/66	3624/3636
	*g*^*p*^_*g*	21	67/73	56/59	3622
	*pl*^*d*^	18	0	180	3648
	*g*^*p*^_*t*	2	70	165	3632
**3 b**	*pl*^*d*^	34	0	180	3644
	*g*^*d*^_*g*	27	15	73	3621
	*g*^*p*^_*chel*	17	62	68	3634
	*perp*_*t*	13	85	172	3625
	*g*^*p*^_*g*	10	68	61	3620
**4 b**	*g*^*d*^_*g*	32	14/15	74/75	3621/3623
	*pl*^*d*^	24	0/1	179/180	3644/3645
	*g*^*p*^_*chel*	21	61/63	68/70	3614/3638
	*g*^*p*^_*g*	14	68/69	61/62	3620/3622
	*perp*_*t*	9	71/82	167/172	3626/3630
**5 b**	*g*^*p*^_*g*	34	67	62	3621
	*g*^*d*^_*g*	30	12/13	76/77	3623
	*pl*^*d*^	28	0	180	3645
	*g*^*p*^_*chel*	4	61	69	3632
	*g*^*p*^_*t*	4	71	166	3630
**6 b**	*g*^*d*^_*g*	35	9	79	3626
	*pl*^*d*^	27	0	180	3643
	*g*^*p*^_*chel*	13	61	72	3633
	*g*^*p*^_*t*	13	72	168	3629
	*g*^*p*^_*g*	12	67	65	3622
**7 b**	*g*^*p*^_*chel*	29	56/61	63/67	3601/3635
	*g*^*d*^_*t*	25	17	177	3631
	*g*^*p*^_*g*	22	58/69	55/60	3606/3621
	*g*^*d*^_*g*	18	15	84	3610
	*g*^*p*^_*t*	6	62/72	165/169	3619
**8 b**	*g*^*p*^_*g*	80	73	63	3623
	*g*^*p*^_*chel*	10	63	66	3631
	*g^d^t*	10	65	179	3625
**9 b**	*g*^*p,d*^_*chel*	83	70	65	3632
	*gt*	16	63/78	163/170	3626
	*g*^*p,s*^_*g*	1	62	67	3619

[a] See Figure 4 for conformer definitions. When relevant, *proximal*/*distal*, *anti*/*syn* and *E*/*Z* conformations are grouped together. The detailed computed relative free energies and conformer populations are provided in the Supporting Information (Table S1). [b] Scaled by 3616/3972=0.91, the ratio between the calculated and the experimental *ν*_OH_ value for benzylalcohol *g*_*g* conformer.

**Table 4:** Calculated populations *p*_i_ of the conformers of difluorinated benzyl alcohol derivatives (1 c–7 c) in CCl_4_ medium at the IEFPCM-MP2/6-311++G(2d,p) level of theory. Optimised dihedral angles *ϕ* (C_*ortho*_C_*ipso*_C_α_O) and *χ* (C_*ipso*_C_α_OH) characterising the hydroxyl moiety orientation and *ν*_OH_ stretching frequencies (IEFPCM-MPWB1 K/6-31+G(d,p))^[a]^

Compound	Conformer	*p*_i_ [%]	ϕ [°]	χ [°]	*ν*_OH_ [cm^−1^]^[b]^
**1 c**	*g*_*chel*	70	60	67	3618
	*perp*_*t*	30	89	180	3625
**2 c**	*perp*_*t*	40	93/94	178/179	3624
	*g*^*p*^_*chel*	36	58/61	67/69	3628/3635
	*g*^*d*^_*chel*	24	59/63	66/69	3626/3636
**3 c**	*g*^*p*^_*chel*	33	62	70	3620
	*g*^*d*^_*chel*	29	59	68	3618
	*perp*_*t*	39	92	178	3625
**4 c**	*g*^*p*^_*chel*	40	60/63	70/72	3618/3633
	*g*^*d*^_*chel*	25	59/62	68/70	3628/3637
	*perp*_*t*	35	89/91	179/180	3624
**5 c**	*perp*_*g*^*−*^	46	85	71	3625
	*perp*_*t*	29	92	178	3624
	*g*^*p*^_*chel*	14	64	72	3624
	*g*^*d*^_*chel*	12	60	70	3631
**6 c**	*perp*_*t*	48	87/89	178/179	3624
	*g*^*p*^_*chel*	31	63	74	3619/3633
	*g*^*d*^_*chel*	20	60	72/73	3625
**7 c**	*g*_*chel*	48	53/65	66/68	3612
	*g*_*g*^*−*^	41	56	62	3623
	*g*_*t*	11	58	177	3618

[a] When relevant, *proximal*/*distal*, *anti*/*syn* and *E*/*Z* conformations are grouped together. The detailed computed relative free energies and conformer populations are provided in SI (Table S1). [b] Scaled by 3616/3972=0.91, the ratio between the calculated and the experimental *ν*_OH_ value for benzylalcohol *g*_*g* conformer.

#### The Various Substituted Benzyl Alcohol Conformations

It should be noted that additional *gauche* and *planar* conformations can be distinguished when substitution in the *ortho* and/or *meta* position of the phenyl ring occurs. The *proximal* and *distal* conformers are therefore defined (Figure 4 (c)) when the hydroxyl group is oriented towards or at the opposite side of the substituent (with the *ortho* substitution prevailing over *meta* substitution, and with *o-*fluorine prevailing over *o-*alkyl groups). With *o*,*o′*-difluorination, *distal*/*proximal* refers to the position of the *meta* substituent. We also distinguish a *g*^*p*^_*chel* conformation from a *g*_*g* conformation (Figure 4 (c)). In this conformation, a short H⋅⋅⋅F distance can be measured (see below). Finally, for compounds with *m-*OMe or *m-*OCF_3_ substituents, additional conformers occur depending on the position of the methyl/trifluoromethyl groups relative to the CH_2_OH group (see the Supporting Information for all structures). For simplicity, the conformers involving rotation along the Ar–OMe or Ar–OCF_3_ bonds are grouped together in Tables 2–4; a full account is provided in the Supporting Information.

#### Conformational Analysis of Substituted Benzyl Alcohols

Within the series of nonfluorinated compounds **1 a**–**9 a** (Table 2), two or three of the various conformations evoked above are found, depending on the studied structure. In all cases, the *g*_*g* is systematically calculated to be the most populated conformation, though it is the absolute energetic minimum only for **1 a**, **4 a**, **6 a**, **7 a** and **9 a**. The other compounds (**2 a**, **3 a**, **5 a** and **8 a**) show a *planar* geometry as the most stable conformation. Our calculations confirm therefore that the *g*_*g* conformation is by far the most (in many cases even the only) populated of the possible *gauche* conformations. A *perpendicular* conformation is seen for **9 a** only.

The computed frequencies of the *ν*_OH_ stretching vibrations show that all the *g*_*g* conformers absorb at a lower frequency than the *pl*, *g*_*t* and *perp* forms. Therefore, the observed lower frequency absorption band was attributed to the *g*_*g* conformer, with the higher frequency band containing the possible contribution of the other conformers. This analysis is in line with a previous attribution by Visser.[22a] No further discrimination can be achieved between the other conformers because of the close values of their IR absorptions. Note that a scale factor has been applied to all computed *ν*_OH_ values to compare easily the experimental and the calculated *ν*_OH_ values for the benzyl alcohol *g*_*g* conformer.

Within the series of *o*-monofluorinated compounds **1 b**–**6 b** (Table 3 and Table S1 in the Supporting Information), the most abundant conformation is *gauche*, despite the *planar* structure is slightly more stable than the most stable *gauche* conformer, generally by approximately 1 kJ mol^−1^. This contrasts with a previous study dealing with 2-fluorobenzyl alcohol **1 b**, for which the *planar* conformation was not identified as a major conformer by MW spectroscopy and MP2 calculations.[24] For the *o-*monofluorinated secondary benzyl alcohol **7 b**, the planar conformation does not feature in the low-energy conformational landscape. In its main low-energy structures, the CHOH–CH_3_ bond is perpendicular to the plane of the phenyl ring, similar to the major ethyl benzene conformation and as found previously in the gas phase by MW spectroscopy.[25] In addition, approximately 10 % of the conformers have both the methyl and the hydroxyl groups in the *gauche* position. It is interesting to note that, apart from the *chelated* conformer, there is little preference for fluorine position: the *proximal* and *distal* populations are not significantly different. For the *o*-fluoro-*o*-alkyl substrates **8 b** and **9 b**, the calculations predict only *proximal gauche* conformations. Presumably, steric hindrance is also a significant factor for **8 b**–**9 b**. In all cases, the *gauche* conformations overwhelmingly display a χ-angle of approximately 60° (i.e., *g*_*g*), whereas *pl* and *perp* conformations display a χ-dihedral angle of 180°.

Notably, all the planar conformations have a *distal* orientation, with a χ-angle of approximately 180°. *Proximal planar* conformations, with *χ*=180°, are not stabilised, presumably because of a repulsive interaction between the O and F atoms. From an IMHB perspective, one would expect to observe *proximal planar* conformations, with *χ*=0°. However, a geometrical relaxation systematically leads to a *gauche* conformation (*g*^*p*^_*chel*), for which a short H⋅⋅⋅F distance can be measured. Within series **b**, this distance ranges from 2.166 to 2.317 Å; that is, 10 to 16 % shorter than the sum of fluorine and hydrogen van der Waals radii.[27] The contributions of this conformation to the whole population vary from 4 % in **4 b** (with the *m*-trifluoromethoxy substituent) to almost 30 % in **7 b** (α-methylbenzylalcohol). In compound **8 b** (*o-*methyl group), the chelated form represents only 10 % of the population. Conversely, in **9 b** (*tert*-butyl substituent), *g*^*p*^_*chel* (83 %) is significantly favoured in comparison with the other conformations, probably because of the steric hindrance induced by this bulky substituent.

The computed frequencies of the *ν*_OH_ stretching band are similar to those of series **a**. Indeed, the *pl* conformations are predicted to absorb at approximately 3640 cm^−1^ and the *g*_*g* forms at approximately 3620 cm^−1^. In addition, the *ν*_OH_ stretching vibration of the *g*^*p*^_*chel* conformers absorb at 3630 cm^−1^. This can explain the shape of the experimental *ν*_OH_ bands, which display broad envelops with no clear maxima, as opposed to three distinguishable bands.

The conformational features of series **c**, with two *ortho*-fluorine atoms, eventually appear to be the simplest (Table 4). Consistent with the existence of only *distal planar* conformations for which there is a fluorine atom in the *o*-position, with *o*,*o′*-difluorination, no *planar* conformations are observed at all. The electronic repulsion between the lone pairs of the oxygen and fluorine atoms is expected to repel the hydroxyl group out of the aromatic plane. Hence, only *gauche* and *perpendicular* conformations are found, the energetically favoured one depending on the chemical nature of the substituent. In this series, the proportion of the OH⋅⋅⋅F *g*^*p*^_*chel* forms is much more important than in the **b** series (e.g., from 25 % in **5 c** to 70 % in **1 c**). It is also interesting to compare the relative population of the *proximal* and *distal chelated* conformations. Indeed, from **2 c** to **6 c**, the *g*^*p*^_*chel* conformers are slightly, but systematically, preferred over the *g*^*d*^_*chel* conformers, indicating a difference in IMHB accepting capacity of the two *ortho*-fluorine atoms. Finally, it is noteworthy that, in most cases in the **c** series, the *gauche* conformations are systematically *chelated*, but **7 c** also shows *g*_*g*^*−*^ and *g*_*t* conformers.

The *ν*_OH_ stretching vibrations for the various conformations of a given compound in series **c** are computed at closer wavenumbers than in series **a** and **b** (from 3612 to 3637 cm^−1^ instead of 3606 to 3648 cm^−1^). This suggests that these conformations absorb at approximately the same frequencies in the 2,6-difluoro derivatives, in agreement with the narrow bands observed experimentally, contrasting with the broad bands obtained for nonfluorinated and monofluorinated compounds.

#### AIM and NCI Analysis

The presence of intramolecular interactions, including a possible OH⋅⋅⋅F IMHB interaction, was investigated by AIM analysis. However, in some cases that have been described previously, the AIM theory has been shown to fail in identifying any bond-critical point (BCP) for an IMHB, whereas other theoretical and experimental features were consistent with the occurrence of such an interaction.[28] We have therefore complemented the AIM calculations by conducting an NCI analysis.

**Figure 5:**
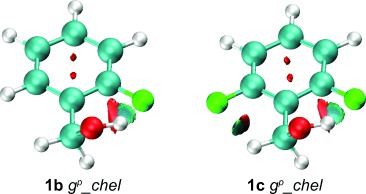
NCI isosurface plots of *g*^*p*^_*chel* benzyl alcohol conformations of 1 b,c drawn with a reduced density gradient (RDG) value of 0.6 and the blue (attractive) green (van der Waals) red (repulsive) values ranging from −0.02 to 0.01 a.u. An attractive OH⋅⋅⋅F contribution is observed in 1 b, and a weak additional C_α_H⋅⋅⋅F interaction is found in 1 c.

The difference in population between chelated and nonchelated *gauche* conformers for series **b** and **c** invited analysis. It was found that, for the relevant *g*^*p*^_*chel* conformations of these compounds, AIM and NCI analyses do confirm the occurrence of an intramolecular OH⋅⋅⋅F hydrogen bond. The electron density at the BCP, ρ_b_, ranging from 0.010 to 0.015 e bohr^−3^, and the positive value of the Laplacian, ∇^2^ρ_b_, are consistent with an IMHB between the fluorine and the hydroxyl moieties for *g*^*p*^_*chel* conformations of the 2-fluorobenzyl alcohols (Table S2). For the 2,6-difluorinated benzyl alcohols, no significant difference between ρ_b_ and ∇^2^ρ_b_ is found with respect to series **b**, suggesting that the OH⋅⋅⋅F interaction strength is similar for the 2-fluoro- and 2,6-difluorobenzyl derivatives. Nevertheless, comparing the *proximal* and *distal* conformers, ρ_b_ is generally found to be slightly higher when the IMHB involves the 6-fluoro (*distal*) rather than the 2-fluoro (*proximal*) atom. This suggests a slightly stronger interaction with the 6-fluorine atom. Moreover, the NCI analysis also shows, for the 2,6-difluorinated derivatives, that an additional C_α_H⋅⋅⋅F attractive contribution occurs, as illustrated in Figure 5 with the example of the **1 c**­ *g*_*chel* conformer. This extra stabilising interaction is clearly not possible for the chelated monofluorinated benzyl alcohols. In addition, with two fluorine atoms in the *ortho* position in series **c**, the *g*_*g* conformations appear as *g*_*chel* conformations, to optimise the attractive OH⋅⋅⋅F and C_α_⋅⋅⋅F interactions and minimise the repulsive O⋅⋅⋅F interactions. This may explain the significant difference in population of the *g*^*p*^_*chel* structure between series **b** and **c**.

An estimation of the HB energy (*E*_HB_), based on the potential energy density *V_b_* at the BCP, has been proposed previously[29] and was found to be 12 kJ mol^−1^ in the case of CH⋅⋅⋅O IMHBs.[30] In the compounds under study, it appears that the energies of the IMHBs occurring between the fluorine and the hydroxyl groups are slightly larger, ranging from 12 to 19 kJ mol^−1^ (Table S2). An examination of the computed *E*_HB_ values for the various derivatives does not reveal any general trend indicating that a significant increase of HB energy occurs from monofluorinated (series **b**) to difluorinated (series **c**) benzyl alcohols. Nevertheless, comparing the *proximal* and *distal* conformers in series **c**, the HB energy values are generally found to be slightly higher when the IMHB involves the 6-fluoro (*distal*) rather than the 2-fluoro (*proximal*) atom. This suggests a slightly stronger interaction with the 6-fluorine atom.

**Figure 6:**
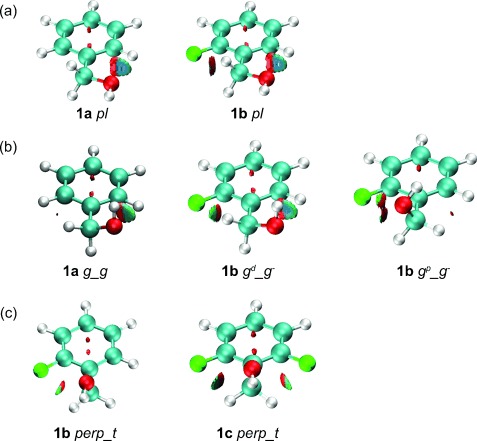
NCI isosurface plots of benzyl alcohols 1 a–c drawn with a reduced density gradient (RDG) value of 0.6 and the blue-green-red values ranging from −0.02 to 0.01 a.u. The *pl* and some *g*_*g* conformations show an attractive CH⋅⋅⋅O contribution, whereas a weak CH⋅⋅⋅F interaction is found in the *perp* and other *g*_*g* conformers.

In fact, many additional intramolecular interactions are revealed by the NCI analysis occurring besides or instead of the OH⋅⋅⋅F interaction, which may provide insight into how the different conformations are stabilised (Figure 6). For example, in the *planar* conformation of **1 a** and **1 b** (Figure 6 (a)), an attractive C_*ortho*_H⋅⋅⋅O interaction is found (Table S2), with a concomitant C⋅⋅⋅F repulsive contribution for **1 b**. For a hypothetical *pl*^*p*^ conformation, the NCI analysis reveals a rather large F⋅⋅⋅OH repulsion, which may explain why there is no *pl*^*p*^ conformation in series **b**, and no *planar* conformation in series **c**. For the *gauche* conformers (Figure 6(b)), an attractive C_*ortho*_H⋅⋅⋅O interaction similarly stabilises the *g*_*g* conformation. In addition, for **1 b**, the *ortho*-fluorination now provides a weak attractive van der Waals C_α_H⋅⋅⋅F interaction (for the *distal gauche*). Conversely, the **1 b**­ *g*^*p*^_*g* structure is destabilised by an O⋅⋅⋅F repulsion. Weak van der Waals C_α_H⋅⋅⋅F interactions also occur in the *perpendicular* forms of **1 b** and **1 c** (Figure 6(c)). These extra intramolecular interactions can also provide an explanation as to why the *g*_*t* conformers rarely occur in the various compounds. It is actually often found that starting from such a conformer, relaxation to a planar conformation to accommodate a stabilising CH⋅⋅⋅O interaction, or to a perpendicular conformation to minimise the O⋅⋅⋅F repulsion and favour van der Waals C_α_H⋅⋅⋅F interactions, occurs.

In most, if not all cases, there are a multitude of attractive and repulsive effects operating, and it is difficult to explain population differences between rotamers based on simple comparisons of interactions.

#### NBO Calculations

A further analysis of the conformational preferences of benzyl alcohol derivatives was carried out through NBO calculations. Only the benzyl, *m*-fluorobenzyl and *m*-nitrobenzyl alcohol series with zero, one, and two *ortho*-fluorines (**1 a**–**c**, **3 a**–**c**, **6 a**–**c**) were considered, each having zero, an intermediate, and a large *m*-substituent effect, respectively. The main *E*^(2)^_n→σ*_ interaction energies are gathered in Table S3 in the Supporting Information.

Considering the conformations exhibiting an OH⋅⋅⋅F IMHB, the interaction energies between the n_F_ fluorine lone pairs and the σ*_OH_ antibonding orbital are rather small (ca. 4 kJ mol^−1^), and do not fundamentally differ from the monofluoro to the difluorobenzyl alcohols. A slight increase of the interaction energies is observed when the chelation occurs with the 6-fluoro substituent rather than with the 2-fluoro substituent. These trends are in reasonable agreement with the electron densities ρ_b_ and *E*_HB_ calculated at the BCP, and further clarify why the chelated structures are not dominant in monofluorinated benzyl alcohols.

For the *planar* and *perpendicular* conformations, the n_O_→σ*_C1−Cα_ interactions are rather weak, with *E*^(2)^_n→σ*_ of 6–8 kJ mol^−1^. In return, hyperconjugation occurs between the σ_OH_ and σ_C1−Cα_ orbitals (ca. 13 kJ mol^−1^ for σ_OH_→σ*_C1−Cα_ and 8 kJ mol^−1^ for σ_C1−Cα_→σ*_OH_). In addition, the weak n_O_→σ*_=CH_ interaction stabilises the *planar* forms of series **a** and **b** slightly (ca. 3 kJ mol^−1^), and an energetically equivalent n_F_→σ*_Cα−O_ interaction also occurs in series **b**. Clearly, the aromatic system is also available to provide a stabilising interaction with the hydroxyl moiety. The charge transfer from the π bonding orbitals to the σ*_Cα−O_ antibonding orbital shows the highest contribution (up to 30 kJ mol^−1^), smaller σ_Cα−O_→π*_C=C_, σ_Cα−O_→σ*_C=C_ and σ_C=C_→σ*_Cα−O_ contributions being systematically found irrespective of the considered conformation.

For the *gauche* conformations, the n_O_→σ*_C1−Cα_ interaction is significant, with *E*^(2)^_n→σ*_ of 30 to 40 kJ mol^−1^ in comparison with the *planar* and *perpendicular* conformations. This may explain the significant preference of the *g*_*g* conformation over the corresponding *g*_*t* conformation (see above). In addition, the π_C=C_→σ*_Cα−O_ interactions are also significant, but, interestingly, only for the fluorinated derivatives (*E*^(2)^_n→σ*_ of 20 to 30 kJ mol^−1^ for series **b**, **c**, but <10 kJ mol^−1^ for series **a**). In summary, numerous hyperconjugative interactions occur in the different conformers of fluorinated benzyl alcohols. If an OH⋅⋅⋅F IMHB indeed appears in some of the *gauche* conformers, it is clearly not a driving force for the conformational preference, with the n_O_→σ*_C1−Cα_, σ_OH_→σ*_C1−Cα_, π_C=C_→σ*_Cα−O_ interactions being at least of the same order of magnitude.

#### Main Conformational Features

To sum up this section, it is shown that the conformational preferences adopted by benzyl alcohols are significantly influenced by the presence of fluorine atom(s) in the *ortho* position. Without any fluorine, *g*_*g* and *pl* conformers represent 80 to 100 % of the relative populations in series **a**, with an attractive C_*ortho*_H⋅⋅⋅O interaction stabilising these conformations. An OH⋅⋅⋅F IMHB conformer, from 4 to 83 %, appears in series **b** with the presence of one fluorine atom, decreasing the relative population of the *g*_*g* conformers, the *pl* population being almost unchanged. The *g*_*chel* and *perp* structures represent 100 % of the population in most difluorinated benzyl alcohols. The stabilisation of the *perp* conformers is due to attractive C_α_H⋅⋅⋅F contacts occurring with both *ortho-*fluorine atoms, whereas an OH⋅⋅⋅F IMHB with one fluorine atom and a C_α_H⋅⋅⋅F interaction with the second fluorine concomitantly stabilise the *g*_*chel* structures. The second case is almost systematically preferred over the first.

### HB Acidity of Benzyl Alcohols

#### Experimental HB Acidity Measurements

**Scheme 1:**
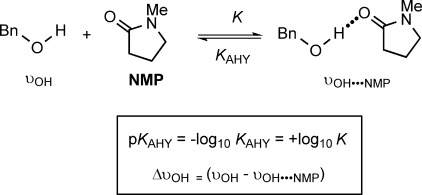
Experimental determination of the HB-donating capacity of the benzyl alcohol derivatives.

The HB-donating capacity of the alcohols was determined by using an IR method through complexation with a standard HB acceptor, *N*-methyl-2-pyrrolidinone (NMP) in CCl_4_ at 25 °C (Scheme 1).[31] The decrease in intensity of the *ν*_OH_ band with increasing amount of NMP was measured, as well as the actual frequency shifts, Δ*ν*_OH_, resulting from complexation with NMP in relation to the corresponding free *ν*_OH(2)_ band. The former allows determination of the equilibrium constant of the reaction, expressed as the thermodynamic HB acidity value *K*_AHY_, and the latter is an indication of the relative strength of the hydrogen bond that is formed. Considering homogeneous families of compounds that do not exhibit additional specific effects (such as steric effects or IMHB), these two experimental parameters are generally well correlated, with Δ*ν*_OH_ then corresponding to a spectroscopic HB scale.[31]

The frequency shifts, Δ*ν*_OH_, the measured p*K*_AHY_, and the corresponding free energies of complexation, Δ*G*_AHY_, are gathered in Table 1. An energetic range of approximately 7 kJ mol^−1^ (1.3 p*K* units) is covered by the current data set.

#### Measurement of the Equilibrium Constants (K_AHY_)

The increase of the HB acidity, p*K*_AHY_, for the nonfluorinated benzyl alcohols (**1 a**–**6 a**) follows the increase in the electron-withdrawing substituent effects of R (H<MeO<F<CF_3_<OCF_3_<NO_2_) in the *meta* position, σ_m_.[32] Therefore, from the unsubstituted benzyl alcohol (1.03) to 3-nitrobenzyl alcohol (1.79), the HB acidity increase represents 4.3 kJ mol^−1^. With the addition of one methyl group in the *ortho*- (**8 a**) or in the α-position (**7 a**), a slight decrease in p*K*_AHY_ is measured (0.2 or 0.4 kJ mol^−1^, respectively).

**Figure 7:**
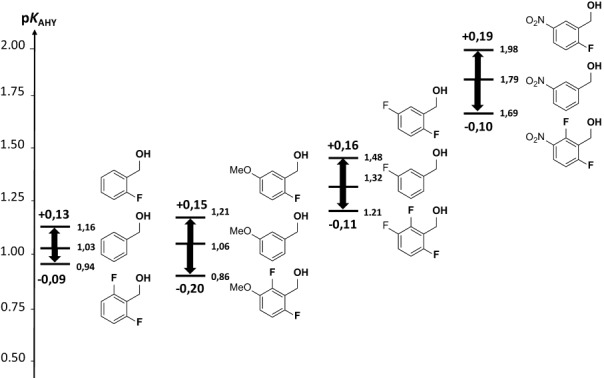
Repartition of the p*K*_AHY_ acidity of substituted benzyl alcohols upon *ortho-*mono- and difluorination.

Similar trends are observed (Figure 7) in the *ortho-*monofluorinated and *o*,*o′-*difluorinated series **b** and **c**, with respective energetic ranges of 5.3 and 4.2 kJ mol^−1^. More interestingly, irrespective of the nature of the *meta-*substituent in series **b**, an increase of HB acidity values (between 0.13 and 0.24 p*K* units, from 0.5 to 1.4 kJ mol^−1^) is measured upon monofluorination with respect to the nonfluorinated counterpart in series **a**. The fluorine atom plays its role of electron-withdrawing substituent, decreasing the electron density around the hydroxyl moiety. With a p*K*_AHY_ value of 1.98, 2-fluoro-5-nitrobenzyl alcohol (**6 b**) is the strongest HB donor in the current experimental data set.

Conversely, further fluorination at the second *ortho*-position does not lead to a further increase of HB acidity (series **c**), highlighting a different behaviour. Indeed, a significant decrease is measured of 0.22 to 0.35 p*K* units in comparison with series **b**, rendering the p*K*_AHY_ values even weaker than for the nonfluorinated benzyl alcohols.

The conformational analysis has shown that the OH⋅⋅⋅F IMHBs are not stronger in the difluorinated than in the monofluorinated derivatives, but rather that the chelated forms allow an optimal accommodation of the attractive OH⋅⋅⋅F and C_α_H⋅⋅⋅F and the repulsive O⋅⋅⋅F interactions, increasing their relative populations (compare the populations of *g*^*p,d*^_*chel* conformers in **b** and **c** derivatives in Tables 3 and 4). This suggests that the decrease of HB acidity in series **c** could originate in the higher propensity of difluorinated derivatives to be intramolecularly chelated. A rational tuning of HB acidity can therefore be realised in such benzyl alcohols by choosing monofluorination rather than difluorination, or vice versa.

A similar evolution is observed for the α-methylbenzyl alcohols **7**, for which an expected HB acidity increase is measured (+0.09) upon monofluorination, whereas the presence of a second fluorine atom has a dramatic weakening effect (−0.35).

Interestingly, a decrease of HB acidity is observed upon fluorination in the case of compounds **8**, rather than an increase, as found for the monofluorinated benzyl alcohols. The OH⋅⋅⋅F IMHB contributes to the HB acidity decrease, but not in a higher proportion than in the other monofluorinated benzyl alcohols because the relative population of the chelated forms are of the same order of magnitude (see below).

#### Measurement of the Frequency Shifts (Δν_OH_)

**Figure 8:**
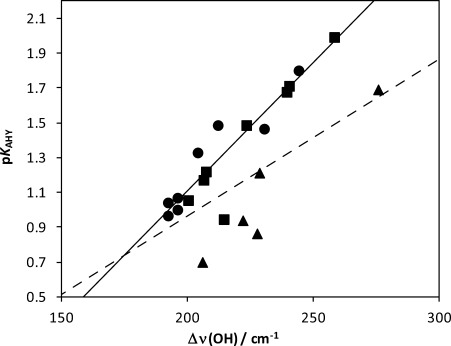
Plot of p*K*_AHY_ versus Δ*ν*(OH) frequency shift for benzylalcohols (square), *o-*fluorobenzylalcohols (circle) and *o*,*o′*-difluorobenzylalcohols (triangle). The whole series c and compound 8 b, are significant outliers from the calibration line (full line) established with series a and b. The dashed line was previously established for a series of hydroxyl compounds.[31]

The highest values of the frequency shifts, upon complexation with NMP, are measured with the 2,6-difluoro derivatives, compared with 2-fluoro and finally nonfluorinated benzyl alcohols. This trend would suggest that the ability of the studied compounds to act as HB donor with an external HB acceptor should increase from series **a** to series **c**. However, as illustrated in Figure 8, the p*K*_AHY_/Δ*ν*(OH) correlation breaks down for the difluorinated benzyl alcohols, with a systematic undervaluation of the experimental HB acidity for the observed IR shift. This illustrates that p*K*_AHY_ and Δ*ν*_OH_ measure two different characteristics: the former is the equilibrium with an external acceptor, the latter is the strength of the hydrogen bond formed. The difluorinated benzyl alcohols lead to the strongest HBs with the acceptor, but the equilibrium reaction with NMP is disturbed by IMHB with the fluorine atoms. Compound **8 b**, with its *o*-methyl substituent, is also a significant outlier, probably for steric reasons. In other words, these downward deviations from the p*K*_AHY_/Δ*ν*(OH) correlation line, observed in a very homogeneous set of benzyl alcohols, are a clear indication of a significant amount of IMHB conformations for the corresponding compounds.

Interestingly, the dashed line in Figure 8 corresponds to the calibration line established for a series of (non-benzylic) hydroxyl-containing compounds in which an upward deviation of the benzyl alcohol **6 a** remained unexplained.[31] It is now rationalised because the benzyl alcohol family finally does not exactly fit to the hydroxyl series.

#### Theoretical HB Acidity Estimation

The electrostatic potential *V*_α_(*r*), as proposed by Kenny,[15] has recently been shown to be an appropriate descriptor to estimate the HB acidity of hydroxyl compounds[31] including fluorohydrins.[6c] We have shown in fluorinated cyclohexanols that the ability of the hydroxyl group to behave as a HB donor is weakened when an IMHB occurs, and a concomitant decrease of the *V*_α_(*r*) value is observed. It remained to be seen whether the flexible fluorinated benzyl alcohol structures under study could be described accurately by this theoretical descriptor. The initial data sets[6c, 31] were considered in the gas phase at the MPWB1K/6-31+G(d,p) level of theory. The *V*_α_(*r*) value was determined for each conformation, and the predicted p*K*_AHY_ value was then the weighted sum.

Such a treatment leads to a poor correlation between the computed *V*_α_(*r*) descriptor and the experimental p*K*_AHY_ HB acidity. Indeed, predicted values and experimental data differ by −0.31 to 0.22, with a sum square of 0.472 and a standard deviation of 0.152 (not shown).

**Figure 9:**
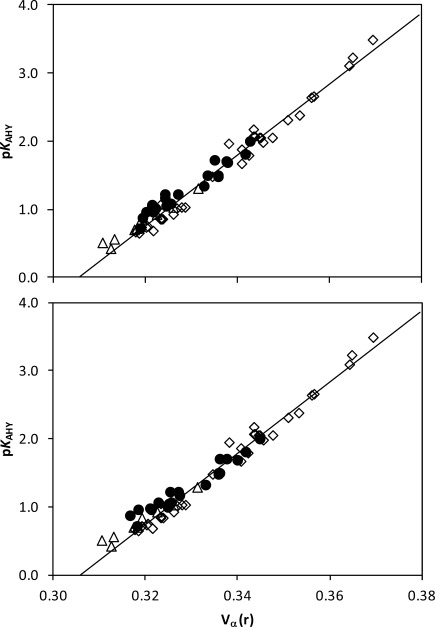
Distribution of the experimental p*K*_AHY_ HB acidity of benzyl alcohols (circle) towards the weighed *V*_α_(*r*) electrostatic descriptor (top: IEF-PCM/MP2/6-311++G(2d,p)//MPWB1K/6-31+G(d,p) level, bottom: IEF-PCM/MPWB1K/6-31+G(d,p) level). The calibration line between these two parameters was previously established with a series of alcohols and phenols (diamond) and of fluorohydrins (triangle).

Therefore, a significant improvement of the theoretical methodology is clearly necessary to obtain useful predicted values. At first, such an improvement may be obtained by including solvation effects in the calculations. Indeed, for a given compound, the population of the different conformers can change significantly in CCl_4_, hence impacting on the weighed *V*_α_(*r*) value, and ultimately the predicted p*K*_AHY_ values (Table 5). The statistics are actually only slightly improved by using either the IEFPCM or the SMD (data not shown) continuum models, with the sum squares decreasing to 0.325 and 0.403, and the standard deviations to 0.127 and 0.142, respectively. Notably, the relative population of the OH⋅⋅⋅F chelated conformers of the 2,6-difluorinated derivatives are systematically smaller at the MP2/6-311++G(2d,p) level with respect to the MPWB1K/6-31+G(d,p) level, whereas they are slightly higher for the 2-fluorinated derivatives. As a consequence, the MP2 weighed *V*_α_(*r*) values are larger than the MPWB1K values in series **c**, leading to higher predicted p*K*_AHY_ values. Conversely, the *V*_α_(*r*) values are lowered in series **b** at the MP2 level, and hence the predicted p*K*_AHY_ values are also lowered. A further improvement of the HB acidity prediction is therefore obtained at the IEFPCM-MP2/6-311++G(2d,p)//MPWB1K/6-31+G(d,p) level, with a sum square value of 0.280 and a standard deviation of 0.112. Figure 9 illustrates the improvement of the correlation between the experimental HB acidity and the computed *V*_α_(*r*) descriptor according to the selected level of theory. However, although a statistical improvement is found with MP2 values, the MPWB1K values are to some extent more chemically reliable. Indeed, if the observed decrease of H-bond acidity upon *o*,*o′*-difluorination of the phenyl ring is rather equivalently estimated with both methods, the increase upon *o*-monofluorination is much more properly described at the DFT level. On the contrary, the MP2 method quasisystematically predicts a decrease of p*K*_AHY_, which is clearly opposite to the experimental trend. For this reason, the use of IEFPCM-MPWB1K/6-31+G(d,p) results for the H-bond acidity prediction of fluorobenzylalcohols is recommended.

**Table 5:** Predicted p*K*_AHY_ HB acidity 0of benzyl alcohols, calculated from the weighted *V*_α_(*r*) values, estimated from either MPWB1K or MP2 populations. The difference with the experimental value is given

Entry	MPWB1K/6-31+G(d,p)		MP2/6-311++G(2d,p)	
	p*K*_AHY (calc)_	Δp*K*_AHY_	p*K*_AHY (calc)_	Δp*K*_AHY_
**1 a**	1.04	0.01	1.01	−0.02
**1 b**	1.16	0.00	1.00	−0.16
**1 c**	0.70	−0.24	0.78	−0.16
**2 a**	1.07	0.01	1.06	0.00
**2 b**	1.14	−0.07	1.00	−0.21
**2 c**	0.59	−0.27	0.74	−0.12
**3 a**	1.45	0.13	1.44	0.12
**3 b**	1.60	0.12	1.47	−0.01
**3 c**	1.05	−0.16	1.15	−0.06
**4 a**	1.60	0.14	1.59	0.13
**4 b**	1.69	−0.01	1.55	−0.15
**4 c**	1.20		1.28	
**5 a**	1.61	0.13	1.60	0.12
**5 b**	1.81	0.14	1.70	0.03
**5 c**	1.38		1.47	
**6 a**	1.92	0.13	1.91	0.12
**6 b**	2.07	0.09	1.96	−0.02
**6 c**	1.61	−0.08	1.69	0.00
**7 a**	0.83	−0.13	0.82	−0.14
**7 b**	0.92	−0.13	0.84	−0.21
**7 c**	0.67	−0.03	0.72	0.02
**8 a**	1.03	0.04	0.89	−0.10
**8 b**	0.84	−0.10	0.85	−0.09
**9 a**	0.67		0.63	
**9 b**	0.39		0.41	

#### Rationalisation of HB Acidity Evolution Trends

It is interesting to observe the evolution of the *V*_α_(*r*) descriptor for the different conformations (Table S1). Comparing equivalent conformations, there is a clear trend for the *V*_α_(*r*) values to be higher for the fluorinated compounds than for the nonfluorinated compounds. This is an expected effect of the fluorine electronegativity on its surroundings. The *planar* conformations (which are always *distal* in series **b**) generally have the largest *V*_α_(*r*) values, owing to the emphasised CH⋅⋅⋅O stabilising interaction depleting the electron density around the oxygen atom and hence around the hydroxyl hydrogen. The fluorine electron-withdrawing effect also operates in the *perp* conformers (when available, compounds **1 b**, **3 b**, **4 b**), as revealed by the increased *V*_α_(*r*) values for mono- and difluorinated substrates. On the other hand, as expected, *g*_*chel* conformations show low *V*_α_(*r*) values because of the intramolecular hydrogen bonding.

The observations discussed above would explain the HB acidity increase for the monofluorinated compounds, because the *g*_*chel* conformations are too weakly populated to have a detrimental effect on the HB acidity, and all the other conformers show an increased *V*_α_(*r*) value. In this context, it is interesting to note that the monofluorinated **8 b**, which has no *pl* conformation contribution due to the *o*-methyl group, shows a HB acidity decrease.

For the difluorinated compounds, with the *g*_*chel* conformations being the dominant conformations, their strongly decreased *V*_α_(*r*) values explain their decrease in HB acidity. Furthermore, in this series, the absence of any *pl* conformations (showing high *V*_α_(*r*) values) further exacerbates this decrease of HB-donating ability.

## Conclusions

A series of 25 benzyl alcohol derivatives has been investigated by FTIR measurements and quantum chemical calculations, revealing the following trends in terms of conformational preferences and HB-donating capacities.

The *g*_*g* conformers are found to be the most populated minima within the series of nonfluorinated compounds (series **a**), with significant amounts of *pl* conformers. An attractive C_*ortho*_H⋅⋅⋅O interaction is found to stabilise these conformations. The occurrence of *g*_*t* and *perp* conformations are found to be more marginal.

With the introduction of one fluorine atom in the *ortho*-position (series **b**), additional *g*_*g* conformers appear, identified as *g*_*chel*, showing an OH⋅⋅⋅F IMHB interaction. Their occurrence ranges from 4 to 83 % at the expense of the nonchelated *g*_*g* conformers. The population of the *pl* conformers remains almost the same as in series **a**.

With a second *ortho*-fluorine (series **c**), the *g*_*chel* and *perp* conformations become almost the only populated forms, whereas they were clearly less abundant in series **a** and **b**. It is shown that both structures benefit from one (*g*_*chel*) or two (*perp*) attractive C_α_H⋅⋅⋅F contacts, in addition to the OH⋅⋅⋅F IMHB for the *g*_*chel* conformers.

As a result, the OH⋅⋅⋅F IMHB is not found to be the main driving force in guiding the conformational preferences of 2-fluorobenzyl alcohols. The population of such chelated conformers is significant for 2,6-difluorobenzyl alcohols with the help of a C_α_H⋅⋅⋅F interaction, but in competition with *perp* conformers in which two C_α_H⋅⋅⋅F interactions occur.

An increase of HB acidity is quasi-systematically measured upon monofluorination, because of the electron-withdrawing effect of fluorine. This is nicely illustrated by the increase of the electrostatic potential descriptor *V*_α_(*r*) values; all the conformers contributing to the HB-donating capacity increase, except the *g*_*chel* conformer, but its population is not important enough to have a significant influence on the overall HB-donating capacity.

The tremendous loss of HB-donating capacity upon difluorination, with the corresponding alcohol being an even weaker HB donor than its nonfluorinated counterpart, is less expected. The contribution of the *perp* structures in series **c** would lead to a further increase in HB acidity compared with monofluorinated benzyl alcohols, but this is overcompensated by the large amount of chelated conformers. Indeed, a significant lowering of the computed *V*_α_(*r*) values for these chelated conformers characterises the HB acidity decrease of series **c**.

The modulations of the HB acidity can therefore be easily rationalised by the *V*_α_(*r*) descriptor, by considering its evolution along the conformational profile. Hence, our study provides methodology to either increase or decrease the HB-donating capacity of benzylic alcohols by judicious fluorination.

## Experimental Section

**Chemicals**: Carbon tetrachloride solvent, of spectroscopic grade, was kept for several days over freshly activated 4 Å molecular sieves before use. Commercial *N*-methyl-2-pyrrolidinone (99.5+% purity) was also stored over molecular sieves in the dark to prevent its deterioration. All benzyl alcohols were dried over 4 Å molecular sieves for the liquid compounds and over P_2_O_5_ during their sublimation for the solid compounds.

**Figure 10:**
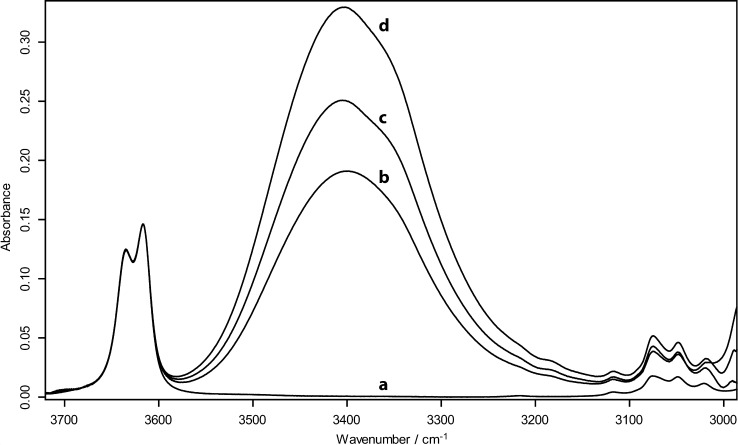
IR spectra of *m*-trifluoromethylbenzyl alcohol (5 a) in the *ν*_OH_ stretching region, a) without and b, c, d) with increasing amounts of *N*-methyl-2-pyrrolidinone. The spectra have been normalised on the free *ν*_OH_ stretching band (3616 cm^−1^) to show the consistency of its profile.

**FTIR spectrometry measurements**: The handling of all chemicals and their CCl_4_ solutions and the filling of the cells for IR measurements were performed in the dry atmosphere of a glove box at RT. IR spectra were recorded in carbon tetrachloride solutions with a Fourier transform spectrometer (Bruker Vertex 70) at a resolution of 1 cm^−1^. An Infrasil quartz cell (l=1 cm path length and thermostatted at 25.0±0.2 °C by Peltier effect regulation) was used for the studies of HB complexation. The HB acidity, p*K*_AHY_, of the benzyl alcohols under study were measured as described recently.[31] The molar absorption coefficients, ε_OH_, required for the equilibrium constant measurements, were calculated for each compound at the frequency of the absorption maxima. It is consequently an apparent ε_OH_ value because the alcohol concentration is distributed over several conformers. This conformation equilibrium is re-established after HB complexation because the shape of this free *ν*_OH_ stretching band is constant, as shown in Figure 10. This apparent value can therefore be safely used to calculate the concentration of free alcohol, and, as a result, the HB equilibrium constant.

### Computational Procedures

All DFT calculations were performed with the D.01 version of the Gaussian 09 program applying default procedures, integration grids, algorithms and parameters.[33] The MPWB1K functional[34] was selected, in combination with the 6-31+G(d,p) basis set, for the conformational study of benzylic alcohols. These compounds appear to be rather flexible, with two main degrees of freedom around the ϕ (C_*ortho*_C_*ipso*_C_α_O) and χ (C_*ipso*_C_α_OH) dihedral angles, and possibly around the *meta*-substituent. In the current work, we have taken into account solvation effects by applying the polarisable continuum solvation model (CCl_4_ as solvent) within the integral equation formalism (IEFPCM). Basso has demonstrated, in the case of flexible 2-halocyclohexanols in dichloromethane, acetone and methanol, that the use of individual spheres for the hydrogen atoms is required to build the molecular cavity in the PCM model,[26] for a proper description of their conformational preferences, the well-known UAHF (United Atom for Hartree–Fock) scheme, with implicit hydrogens, led to results that opposed the NMR experimental trends. Hence, we have used the UFF cavity model,[35] which allows hydrogen atoms to be described explicitly, during each geometry optimisation procedure. The conformational equilibrium of benzyl alcohol derivatives was investigated at the IEFPCM-MPWB1K/6-31+G(d,p) level The vibrational spectrum was computed for each optimised structure to check that there was no imaginary frequencies and to obtain free energies. Single-point calculations were then performed at the IEFPCM-MP2/6-311++G(2d,p) level. The relative populations *p_i_* [Eq. (1)] of the various conformers were hence evaluated from the computed free energies, at the selected levels of theory, through a Boltzmann distribution. The theoretical descriptors were weighted according to these populations.


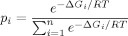
(1)

IMHB interactions were analysed in detail through AIM topological analysis of the MPWB1K/6-31+G(d,p) wave functions with the AIM2000 program.[39] Besides the electron densities ρ_b_ and their Laplacians ∇^2^ρ_b_, the potential energy density *V*_b_ at the BCP is often used to gain additional insights into the strength of a given HB.[36] Indeed, the HB energy can be estimated by using *V*_b_ according to the established relationship in Equation (2):[29]


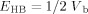
(2)

The NCI topological[13] and NBO[14] analyses of the same wavefunctions were performed with NCIPLOT 3.0[37] and NBO 6.0[38] programs, respectively.

The HB acidity of the compounds under study were evaluated as recommended previously[31] through calculation of the Kenny *V*_α_(*r*) descriptor.[15] It involves calculating the electrostatic potential value along the OH bond at a distance *r*=0.55 Å from the hydroxyl hydrogen atom, at the MPWB1K/6–31+G(d,p) level in vacuo to use the established calibration line [Eq. (3)]:


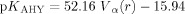
(3)


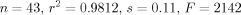


## Supporting Information

As a service to our authors and readers, this journal provides supporting information supplied by the authors. Such materials are peer reviewed and may be re-organized for online delivery, but are not copy-edited or typeset. Technical support issues arising from supporting information (other than missing files) should be addressed to the authors.

miscellaneous_information
