# Supplementary material for: Influence of Fluorination on the Conformational Properties and Hydrogen-Bond Acidity of Benzyl Alcohol Derivatives
Source: Chemistry. 2015 Jun 30;21(32):11462–74. doi: 10.1002/chem.201501171 (PMC4531824; doi:10.1002/chem.201501171)

CHEMISTRY
A European Journal

Supporting Information

Influence of Fluorination on the Conformational Properties and Hydrogen-Bond Acidity of Benzyl Alcohol Derivatives

Elena Bogdan,[a] Guillaume Compain,[b] Lewis Mtashobya,[b] Jean-Yves Le Questel,[a] François Besseau,[a] Nicolas Galland,[a] Bruno Linclau,*[b] and Jérôme Graton*[a]

chem_201501171_sm_miscellaneous_information.pdf

1 Nomenclature applied to distinguish the various benzyl alcohol conformations: 2

2 Detailed conformational analysis results........ 5

3 Detailed results from AIM analysis 11

4 Detailed results of NBO analysis 13

5 Experimental procedures........ 14

5.1 2-Fluorobenzyl alcohol 14

5.2 2-Fluoro-5-methoxylbenzyl alcohol........ 14

5.3 2-Fluoro-5-nitrobenzyl alcohol........ 15

5.4 2,6-difluoro-3-nitrobenzyl alcohol........ 15

6 Spectra of the compounds........ 17

6.1 2-Fluorobenzyl alcohol 17

6.2 2-Fluoro-5-methoxybenzyl alcohol........ 19

6.3 2-Fluoro-5-nitrobenzyl alcohol........ 21

6.4 2,6-difluoro-3-nitrobenzyl alcohol........ 23

1 Nomenclature applied to distinguish the various benzyl alcohol conformations:

CC–CO dihedral angle: φ

CC–OH dihedral angle: χ

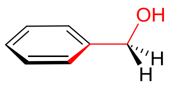

perpendicular (perp)

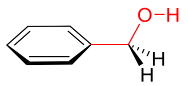

perpendicular_trans
(perp_t)

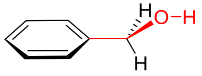

gauche_trans
(g_t)

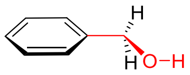

planar_trans
(pl_t)

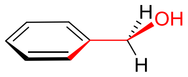

gauche (g)

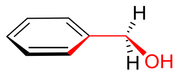

planar (pl)

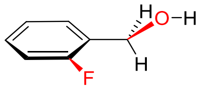

gauche(proximal)_trans
(g[p]_t)

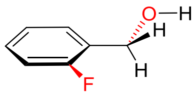

gauche(distal)_trans
(g[d]_t)

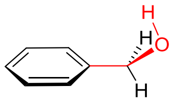

gauche_gauche
(g_g)

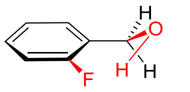

gauche(proximal)_chelated
(g[p]_chel)

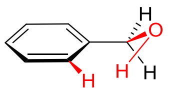

gauche_gauche(2)
(g_g(2))

Two *gauche_gauche* conformations can be distinguished: the so-called *g_g*, and the *g_g(2)*, which is actually never observed owing to the repulsion of the OH and CH groups..

With substitution:
When the orientation of the OH group is towards the *ortho*-substituent, as in **A**, then it is *proximal*, in the other case (**B**), it is *distal*. The *ortho*-substituent has priority over the *meta*-substituent (**C**). With no, or two equal *ortho*-substituents, the relative orientation of the OH-group is referred to the position of the *meta*-substituent (**D**).
See **I** and **II** for examples.

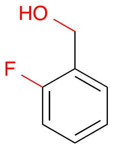

A
proximal (p)

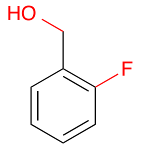

B
distal (d)

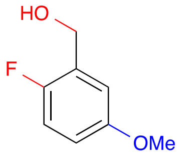

C
proximal
(o > m)

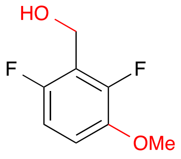

D
distal

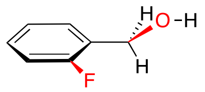

I
proximal (p)
(g[p]_t shown)

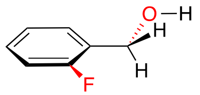

II
distal (d)
(g[d]_t shown)

<u>Substituent *in-plane* conformations</u>

The O–Me group is typically located in the plane of the aromatic ring, due to the presence of the resonance structure **III**. In analogy with amide *E* and *Z* isomers, the *E*/*Z* nomenclature is applied here, cf **E** and **F**.

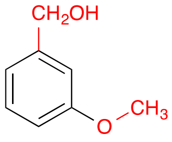

E
Z

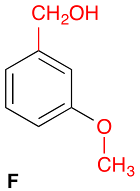

F
E

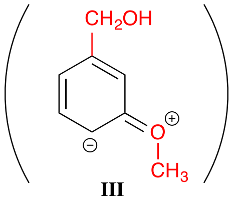

III

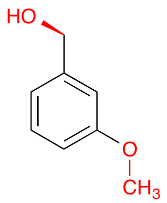

IV
$g^{d,E}$

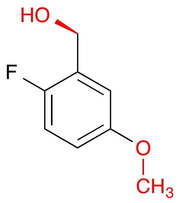

V
$g^{p,E}$

See **IV** and **V** for examples. In **IV**, the alcohol is *distal* with the OMe group, and the OMe conformation is *E*. In **V**, the *ortho*-substituent has priority for the alcohol conformation, but the OMe remains *E.*

<u>Substituent *out-of-plane* conformations</u>

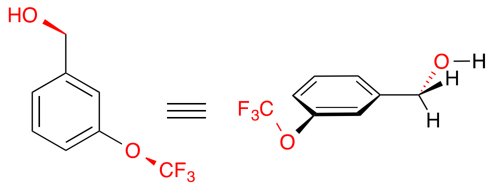

G
distal, syn
$g^{d,s}$

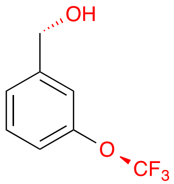

H
proximal, anti
$g^{p,a}$

In contrast to a OMe group, the trifluoromethoxy group is typically located *out-of-plane*, with the O–CF_3 bond perpecdicular to the plane of the aromatic ring. Hence, *syn*/*anti* nomenclature now indicates their position relative to the CH_2OH group, if the latter is in the *gauche* or *perpendicular* conformation: a *syn* conformation is when both are oriented on the same side of the ring, *anti* when different (see **G**, **H**). By convention, the BnOH alcohol orientation is indicated first. Hence, here it is not specified that the CF_3 is perpendicular.

With the bezylic alcohol group in the *planar* conformation, and the trifluoromethyl group in the *perpendicular* conformation, the *syn/anti* nomenclature cannot be used any more. As shown for **I**, the *proximal/distal* nomenclature is required to indicate the relative position of the OH group, and now we do indicate that the CF_3 is *perpendicular* (as opposed to *E* or *Z*).

Finally, when both OH and trifluoromethyl groups are *perpendicular*, it needs to be indicated whether they are *syn* or *anti*. Again, we do indicate that the CF_3 is *perpendicular* (see **J**)

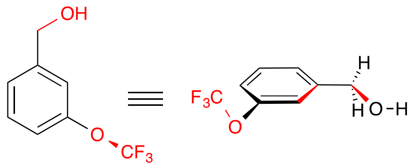

I
proximal, perp
***pl**^{p,perp}*

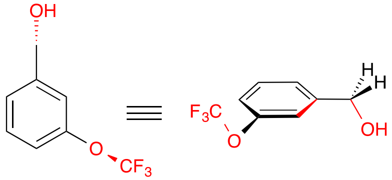

J
perp, anti
***perp**^{a,perp}*

For the trifluoromethyl substituted compounds, conformations can be distinguished where one of the C–F bonds is in the plane of the aromatic ring (*eclipsed*, **K**), or *out-of-plane* with the ring (*perpendicular*, **L**). For the former, it is indicated whether the *in-plane* C–F bond is *distal* or *proximal* to the CH_2OH group. For the latter, it is indicated whether the *out-of-plane* C–F bond is *syn* or *anti* with an *out-of-plane* C–OH bond.

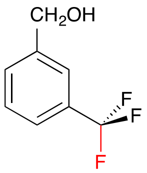

K
eclipsed
(distal)

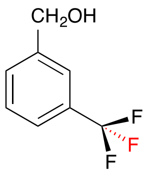

L
perpendicular
(anti or syn)

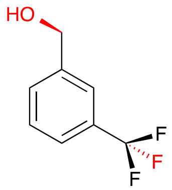

VI
***g**$^{d,p(s)}$*

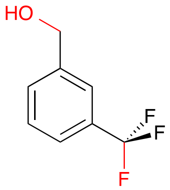

VI
***pl**$^{d,e(d)}$*

For the nitro compounds, :
Though the nitro group is always planar in the non fluorinated and monofluorinated nitrobenzyl alcohol, in the difluoro derivative, it is just out of plane because of the repulsion with the fluorine atom, leading to two distinct conformations. When the C–OH is *out-of-plane*, reference is made with the nitro oxygen nearest to the adjacent fluorine atom. The *syn* descriptor is used for when this oxygen and the C–OH bond are on the same side of the aromatic ring (**M**), and *anti* when on different sides (**N**).,

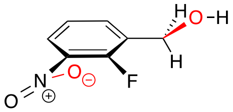

M
syn
***g**^{d,s}*

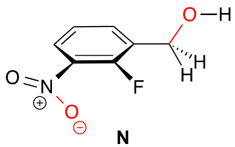

N
anti
***perp**^{a}*

2 Detailed conformational analysis results

Table S1. Absolute (a.u.) and relative Gibbs free energies (kJ mol^{-1}), calculated at the IEF-PCM/MPWB1K/6-31+G(d,p) and IEF-PCM/MP2/6-311++G(2d,p) levels of theory. Electrostatic potential values V_α(r) for each conformer, and weighted value $\bar{V}_\alpha$(r) (in a.u.) and calculated H-bond acidity values pK_{AHY}.

Benzyl alcohol. **1a**

Conformer	IEF-PCM/MPWB1K/6-31+G(d,p)							IEF-PCM/MP2/6-311++G(2d,p)					
	G	Δ*G*	deg.	p_i	V_α(r)	$\bar{V}_\alpha$(r)	pK_{AHY}	*G*	Δ*G*	deg.	p_i	$\bar{V}_\alpha$(r)	pK_{AHY}
g_g^{-}	-346.528669	0.0	2	75.6	0.3234	0.3255	1.04	-344.692635	0.0	2	81.7	0.3249	1.01
pl	-346.623416	1.1	1	24.4	0.3318			-344.691307	2.0	1	18.3		

2-Fluorobenzyl alcohol. **1b**

Conformer	IEF-PCM/MPWB1K/6-31+G(d,p)							IEF-PCM/MP2/6-311++G(2d,p)					
	G	Δ*G*	deg.	p_i	V_α(r)	$\bar{V}_\alpha$(r)	pK_{AHY}	*G*	Δ*G*	deg.	p_i	$\bar{V}_\alpha$(r)	pK_{AHY}
pl^{d}	-445.754669	0.0	1	29.9	0.3351			-444.942350	0.0	1	16.8		
$g^{d}\_g^{-}$	-445.754229	1.2	2	37.5	0.3305			-444.941850	1.3	2	19.8		
g^{p}_chel	-445.753501	3.1	2	17.4	0.3127	0.3278	1.16	-444.941900	1.2	2	20.8	0.3247	1.00
$g^{p}\_g^{-}$	-445.753079	4.2	2	11.1	0.3229			-444.942232	0.3	2	29.6		
perp_t	-445.752144	6.6	2	4.1	0.3259			-444.941454	2.4	2	13.0		

2,6-Difluorobenzyl alcohol. **1c**

Conformer	IEF-PCM/MPWB1K/6-31+G(d,p)							IEF-PCM/MP2/6-311++G(2d,p)					
	G	Δ*G*	deg.	p_i	V_α(r)	$\bar{V}_\alpha$(r)	pK_{AHY}	*G*	Δ*G*	deg.	p_i	$\bar{V}_\alpha$(r)	pK_{AHY}
g_chel	-544.977438	0.0	4	84.4	0.3173	0.3190	0.70	-544.051252	0.0	4	69.9	0.3206	0.78
perp_t	-544.976501	2.5	2	15.6	0.3280			-544.051109	0.4	2	30.1		

3-methoxybenzyl alcohol. **2a**

Conformer	IEF-PCM/MPWB1K/6-31+G(d,p)							IEF-PCM/MP2/6-311++G(2d,p)					
	G	Δ*G*	deg.	p_i	V_α(r)	$\bar{V}_\alpha$(r)	pK_{AHY}	*G*	Δ*G*	Deg.	p_i	$\bar{V}_\alpha$(r)	pK_{AHY}
$pl^{p,E}$	-460.978343	0.0	1	29.9	0.3297			-460.089981	0.0	1	18.3		
$g^{p,Z}\_g^{-}$	-460.977715	1.7	2	24.1	0.3241			-460.089812	0.5	2	30.5		
$g^{p,E}\_g^{-}$	-460.977193	3.0	2	13.9	0.3215			-460.088918	2.8	2	11.8		
$g^{d,E}\_g^{-}$	-460.976873	3.9	2	9.9	0.3211	0.3261	1.07	-460.088779	3.2	2	10.2	0.3260	1.06
$g^{d,Z}\_g^{-}$	-460.976722	4.3	2	8.4	0.3230			-460.088553	3.8	2	8.0		
$g^{p,Z}\_t$	-460.976817	4.0	2	9.3	0.3326			-460.088817	3.1	2	10.6		
$g^{d,Z}\_t$	-460.976636	4.5	2	7.7	0.3311			-460.088489	3.9	2	7.5		
$g^{d,E}\_t$	-460.975831	6.6	2	3.3	0.3294			-460.087611	6.2	2	3.0		

2-fluoro-5-methoxybenzyl alcohol. **2b**

Conformer	IEF-PCM/MPWB1K/6-31+G(d,p)							IEF-PCM/MP2/6-311++G(2d,p)					
	G	ΔG	deg.	p_i	$V_\alpha(r)$	$\bar{V}_\alpha(r)$	pK_{AHY}	G	ΔG	deg.	p_i	$\bar{V}_\alpha(r)$	pK_{AHY}
$pl^{d,Z}$	-560.201386	0.0	1	17.0	0.3366			-559.198699	0.0	1	13.1		
$g^{d,Z}\_g^{-}$	-560.201367	0.1	2	33.2	0.3314			-559.198597	0.3	2	19.8		
$g^{d,E}\_g^{-}$	-560.200760	1.6	2	17.5	0.3283			-559.197858	2.2	2	10.7		
$pl^{d,E}$	-560.200595	2.1	1	7.3	0.3332			-559.197787	2.4	1	5.0		
$g^{p,Z}\_chel$	-560.200247	3.0	2	10.1	0.3108	0.3275	1.14	-559.198211	1.3	2	15.6	0.3247	1.00
$g^{p,E}\_chel$	-560.199928	3.8	2	7.2	0.3098			-559.197622	2.8	2	8.4		
$g^{p,Z}\_g^{-}$	-560.199499	5.0	2	4.6	0.3232			-559.198259	1.2	2	16.4		
$g^{p,E}\_g^{-}$	-560.198799	6.8	2	2.2	0.3209			-559.197160	4.0	2	5.1		
$g^{p,E}\_t$	-560.197900	9.2	2	0.8	0.3240			-559.196420	6.0	2	2.3		

2,6-difluoro-3-methoxybenzyl alcohol. **2c**

Conformer	IEF-PCM/MPWB1K/6-31+G(d,p)							IEF-PCM/MP2/6-311++G(2d,p)					
	G	ΔG	deg.	p_i	$V_\alpha(r)$	$\bar{V}_\alpha(r)$	pK_{AHY}	G	ΔG	deg.	p_i	$\bar{V}_\alpha(r)$	pK_{AHY}
$g^{p,E}\_chel$	-659.526164	0.0	2	40.0	0.3139			-656.367898	0.2	2	29.2		
$g^{d,E}\_chel$	-659.526445	0.9	2	27.9	0.3138			-656.367928	1.8	2	14.9		
$perp^{E}\_t$	-659.418008	2.2	2	16.7	0.3260	0.3170	0.59	-656.366968	0.0	2	31.1	0.3198	0.74
$g^{d,Z}\_chel$	-659.417206	4.3	2	7.2	0.3189			-656.367919	2.9	2	9.7		
$g^{p,Z}\_chel$	-659.523203	4.8	2	5.8	0.3198			-656.367437	3.8	2	6.6		
$perp^{Z}\_t$	-659.521581	6.9	2	2.5	0.3294			-656.367246	3.2	2	8.5		

3-fluorobenzyl alcohol. **3a**

Conformer	IEF-PCM/MPWB1K/6-31+G(d,p)							IEF-PCM/MP2/6-311++G(2d,p)					
	G	ΔG	deg.	p_i	$V_\alpha(r)$	$\bar{V}_\alpha(r)$	pK_{AHY}	G	ΔG	deg.	p_i	$\bar{V}_\alpha(r)$	pK_{AHY}
pl^{p}	-445.755316	0.0	1	28.3	0.3373			-444.942863	0.0	1	27.0		
pl^{d}	-445.754862	1.2	1	17.5	0.3370	0.3334	1.45	-444.942360	1.3	1	15.9	0.3332	1.44
$g^{p}\_g^{-}$	-445.754683	1.7	2	29.0	0.3303			-444.942348	1.4	2	31.3		
$g^{d}\_g^{-}$	-445.754552	2.0	2	25.2	0.3302			-444.942165	1.8	2	25.8		

2,5-difluorobenzyl alcohol. **3b**

Conformer	IEF-PCM/MPWB1K/6-31+G(d,p)							IEF-PCM/MP2/6-311++G(2d,p)					
	G	ΔG	deg.	p_i	$V_\alpha(r)$	$\bar{V}_\alpha(r)$	pK_{AHY}	G	ΔG	deg.	p_i	$\bar{V}_\alpha(r)$	pK_{AHY}
pl^{d}	-544.978877	0.0	1	42.6	0.3406			-544.052147	0.0	1	34.1		
$g^{d}\_g^{-}$	-544.978196	1.8	2	41.4	0.3370			-544.051265	2.3	2	26.8		
g^{p}_chel	-544.976829	5.4	2	9.7	0.3192	0.3364	1.60	-544.050813	3.5	2	16.6	0.3338	1.47
$g^{p}\_g^{-}$	-544.975797	8.1	2	3.3	0.3291			-544.050337	4.8	2	10.0		
$perp\_t$	-544.975719	8.3	2	3.0	0.3313			-544.050553	4.2	2	12.6		

2,3,6-trifluorobenzyl alcohol. **3c**

Conformer	IEF-PCM/MPWB1K/6-31+G(d,p)							IEF-PCM/MP2/6-311++G(2d,p)					
	G	Δ*G*	deg.	p_i	$V_\alpha(r)$	$\bar{V}_\alpha(r)$	pK_{AHY}	*G*	Δ*G*	deg.	p_i	$\bar{V}_\alpha(r)$	pK_{AHY}
g^{p}_chel	-644.193643	0.0	2	41.4	0.3248			-643.153468	0.4	2	32.6		
g^{d}_chel	-644.193592	0.1	2	39.2	0.3229	0.3258	1.05	-643.153344	0.8	2	28.6	0.3276	1.15
perp_t	-644.192926	1.9	2	19.4	0.3334			-634.153628	0.0	2	38.7		

3-trifluoromethoxybenzyl alcohol. **4a**

Conformer	IEF-PCM/MPWB1K/6-31+G(d,p)							IEF-PCM/MP2/6-311++G(2d,p)					
	G	Δ*G*	deg.	p_i	$V_\alpha(r)$	$\bar{V}_\alpha(r)$	pK_{AHY}	*G*	Δ*G*	Deg.	p_i	$\bar{V}_\alpha(r)$	pK_{AHY}
$g^{d,s}$_g^-	-758.687147	0.0	2	18.0	0.3335			-757.458732	0.0	2	25.3		
$pl^{d,E}$	-758.686912	0.6	1	7.0	0.3396			-757.457294	3.8	1	2.8		
$pl^{p,E}$	-758.686857	0.8	1	6.6	0.3396			-757.457311	3.7	1	2.8		
$pl^{p,perp}$	-758.686855	0.8	2	13.2	0.3400			-757.458427	0.8	2	18.3		
$g^{p,a}$_g^-	-758.686795	0.9	2	12.4	0.3339			-757.458393	0.9	2	17.7		
$pl^{d,perp}$	-758.686653	1.3	2	10.7	0.3405			-757.458040	1.8	2	12.2		
$pl^{d,Z}$	-758.686437	1.9	1	4.3	0.3393	0.3363	1.60	-757.456849	4.9	1	1.7	0.3361	1.59
$g^{p,E}$_g^-	-758.686220	2.4	2	6.8	0.3331			-757.456754	5.2	2	3.1		
$g^{d,a}$_g^-	-758.686176	2.6	2	6.4	0.3338			-757.457742	2.6	2	8.9		
$g^{d,E}$_g^-	-758.686005	3.0	2	5.4	0.3329			-757.456593	5.6	2	2.6		
$pl^{p,Z}$	-758.685937	3.2	1	2.5	0.3393			-757.456525	5.8	1	1.2		
$g^{p,Z}$_g^-	-758.685691	3.8	2	3.9	0.3324			-757.456390	6.2	2	2.1		
$g^{d,Z}$_g^-	-758.685372	4.7	2	2.8	0.3326			-757.455957	7.3	2	1.3		

2-fluoro-5-trifluoromethoxybenzyl alcohol. **4b**

Conformer	IEF-PCM/MPWB1K/6-31+G(d,p)							IEF-PCM/MP2/6-311++G(2d,p)					
	G	Δ*G*	deg.	p_i	$V_\alpha(r)$	$\bar{V}_\alpha(r)$	pK_{AHY}	*G*	Δ*G*	deg.	p_i	$\bar{V}_\alpha(r)$	pK_{AHY}
$pl^{d,s}$	-857.911166	0.0	1	12.0	0.3434			-856.562710	0.0	1	11.0		
$g^{d,s}$_g^-	-857.911165	0.0	2	23.9	0.3395			-856.568185	0.2	2	20.1		
$pl^{d,Z}$	-857.910945	0.6	1	9.5	0.3425			-856.567475	2.1	1	4.7		
$pl^{d,a}$	-857.910749	1.1	1	7.7	0.3434			-856.567813	1.2	1	6.8		
$g^{d,a}$_g^-	-857.910628	1.4	2	13.6	0.3403			-856.567458	2.1	2	9.3		
$pl^{d,E}$	-857.910104	2.8	1	3.9	0.3429			-856.566163	5.5	1	1.2		
$g^{p,s}$_chel	-857.909803	3.6	2	5.7	0.3233	0.3380	1.69	-856.567511	2.0	2	9.8	0.3354	1.55
$g^{d,Z}$_g^-	-857.909694	3.9	2	5.0	0.3388			-856.565964	6.1	2	1.9		
$g^{p,a}$_chel	-857.909590	4.1	2	4.5	0.3237			-856.567419	2.2	2	8.9		
$g^{p,E}$_chel	-857.909230	5.1	2	3.1	0.3228			-856.566107	5.7	2	2.2		
$g^{d,E}$_g^-	-857.909038	5.6	2	2.5	0.3396			-856.564993	8.6	2	0.7		
$g^{p,s}$_g^-	-857.908870	6.0	2	2.1	0.3317			-856.567228	2.7	2	7.3		
$g^{p,a}$_g^-	-857.908528	6.9	2	1.5	0.3326			-856.566835	3.8	2	4.8		

$perp^{a}$_t	-857.908293	7.5	2	1.1	0.3342			-856.566611	4.4	2	3.8		
$perp^{Z}$_t	-857.908134	8.0	2	1.0	0.3331			-856.566093	5.7	2	2.2		
$perp^{s}$_t	-857.907951	8.4	2	0.8	0.3339			-856.566295	5.2	2	2.7		
$g^{p,Z}$_chel	-857.907929	8.5	2	0.8	0.3212			-856.564943	8.7	2	0.6		
$g^{p,E}$_g^{-}	-857.907796	8.9	2	0.7	0.3317			-856.565271	7.9	2	0.9		
$g^{p,Z}$_g^{-}	-857.907403	9.9	2	0.4	0.3310			-856.565059	8.4	2	0.7		
$perp^{E}$_t	-857.907009	10.9	2	0.3	0.3335			-856.564472	10.0	2	0.4		

2,6-difluoro-3-trifluoromethoxybenzyl alcohol. **4c**

Conformer	IEF-PCM/MPWB1K/6-31+G(d,p)							IEF-PCM/MP2/6-311++G(2d,p)					
	G	Δ*G*	deg.	p_i	$V_\alpha(r)$	$\bar{V}_\alpha(r)$	pK_{AHY}	*G*	Δ*G*	deg.	p_i	$\bar{V}_\alpha(r)$	pK_{AHY}
$g^{p,s}$_chel	-957.126792	0.0	2	30.2	0.3265			-955.670177	0.00	2	26.7		
$g^{d,a}$_chel	-957.126282	1.3	2	17.6	0.3274			-955.669580	1.57	2	14.2		
$g^{p,a}$_chel	-957.125906	2.3	2	11.8	0.3278			-955.669298	2.3	2	10.5		
$g^{d,s}$_chel	-957.125873	2.4	2	11.4	0.3272			-955.669218	2.5	2	9.7		
$perp^{a}$_t	-657.125755	2.7	2	10.1	0.3360			-955.669879	0.8	2	19.5		
$perp^{s}$_t	-957.125270	4.0	2	6.0	0.3357	0.3287	1.20	-955.669407	2.0	2	11.8	0.3301	1.28
$g^{p,E}$_chel	-957.125171	4.3	2	5.4	0.3270			-955.667996	5.7	2	2.6		
$g^{d,E}$_chel	-957.124610	5.7	2	3.0	0.3252			-955.667163	7.9	2	1.1		
$perp^{E}$_t	-957.124502	6.0	2	2.7	0.3353			-955.668079	5.5	2	2.9		
$g^{p,Z}$_chel	-957.123362	9.0	2	0.8	0.3270			-955.666132	10.6	2	0.4		
$g^{d,Z}$_chel	-957.123262	9.3	2	0.7	0.3263			-955.665902	11.2	2	0.3		
$perp^{Z}$_t	-957.122336	11.7	2	0.3	0.3361			-955.665987	11.0	2	0.3		

3-trifluoromethylbenzyl alcohol. **5a**

Conformer	IEF-PCM/MPWB1K/6-31+G(d,p)							IEF-PCM/MP2/6-311++G(2d,p)					
	G	Δ*G*	deg.	p_i	$V_\alpha(r)$	$\bar{V}_\alpha(r)$	pK_{AHY}	*G*	Δ*G*	deg.	p_i	$\bar{V}_\alpha(r)$	pK_{AHY}
$pl^{d,perp}$	-683.496823	0.0	2	15.3	0.3415			-682.371099	0.6	2	13.2		
$g^{d,d}$_g^{-}	-683.496764	0.2	2	14.3	0.3346			-682.371327	0.0	2	16.8		
$pl^{p,perp}$	-683.496725	0.3	2	13.7	0.3407			-682.371029	0.8	2	12.3		
$g^{p,s}$_g^{-}	-683.496651	0.5	2	12.7	0.3344	0.3365	1.61	-682.371053	0.7	2	12.6	0.3363	1.60
$g^{d,a}$_g^{-}	-683.496614	0.6	2	12.2	0.3347			-682.371112	0.6	2	13.4		
$g^{p,a}$_g^{-}	-683.496613	0.6	2	12.2	0.3345			-682.370965	1.0	2	11.5		
$g^{d,s}$_g^{-}	-683.496591	0.6	2	11.9	0.3348			-682.371079	0.7	2	13.0		
$g^{p,d}$_g^{-}	-683.496166	1.7	2	7.6	0.3346			-682.370519	2.1	2	7.2		

2-fluoro-5-trifluoromethylbenzyl alcohol. **5b**

Conformer	IEF-PCM/MPWB1K/6-31+G(d,p)							IEF-PCM/MP2/6-311++G(2d,p)					
	G	Δ*G*	deg.	p_i	$V_\alpha(r)$	$\bar{V}_\alpha(r)$	pK_{AHY}	*G*	Δ*G*	deg.	p_i	$\bar{V}_\alpha(r)$	pK_{AHY}
$g^{d,a}$_g^{-}	-782.722311	0.0	2	28.6	0.3411	0.3403	1.81	-781.482095	1.1	2	19.8	0.3383	1.70

$pl^{d,p(a)}$	-782.722285	0.1	2	27.8	0.3440			-781.482160	1.0	2	21.2		
$pl^{d,e(d)}$	-782.721863	1.2	1	8.9	0.3440			-781.481728	2.1	1	6.7		
$g^{d,e(d)}$_g^-	-782.721647	1.7	2	14.2	0.3412			-781.481427	2.9	2	9.8		
$g^{p,p(s)}$_g^-	-782.721603	1.9	2	13.5	0.3333			-781.482523	0.0	2	31.2		
$g^{p,p(a)}$_*chel*	-782.719835	6.5	2	2.1	0.3245			-781.480093	6.4	2	2.4		
$g^{p,p(s)}$_*chel*	-782.719726	6.8	2	1.9	0.3244			-781.479995	6.6	2	2.1		
$g^{p,p(a)}$_g^-	-782.719340	7.8	2	1.2	0.3333			-781.480088	6.4	2	2.4		
$g^{p,p(a)}$_*t*	-782.719178	8.2	2	1.0	0.3351			-781.480112	6.3	2	2.4		
$g^{p,p(s)}$_*t*	-782.718947	8.8	2	0.8	0.3351			-781.479892	6.9	2	1.9		

2,6-difluoro-3-trifluoromethylbenzyl alcohol. **5c**

Conformer	IEF-PCM/MPWB1K/6-31+G(d,p)							IEF-PCM/MP2/6-311++G(2d,p)					
	G	Δ*G*	deg.	p_i	$V_\alpha(r)$	$\bar{V}_\alpha(r)$	pK_{AHY}	*G*	Δ*G*	deg.	p_i	$\bar{V}_\alpha(r)$	pK_{AHY}
*perp*_g^-	-881.938683	0.0	2	36.3	0.3346			-878.102718	0.0	2	45.7		
g^d_*chel*	-881.938370	0.8	2	26.1	0.3287	0.3321	1.38	-878.101423	3.4	2	11.6	0.3338	1.47
g^p_*chel*	-881.938252	1.1	2	23.0	0.3287			-878.101598	2.9	2	14.0		
*perp*_*t*	-881.937825	2.3	2	14.6	0.3370			-878.102278	1.2	2	28.7		

3-nitrobenzyl alcohol. **6a**

Conformer	IEF-PCM/MPWB1K/6-31+G(d,p)							IEF-PCM/MP2/6-311++G(2d,p)					
	G	Δ*G*	deg.	p_i	$V_\alpha(r)$	$\bar{V}_\alpha(r)$	pK_{AHY}	*G*	Δ*G*	deg.	p_i	$\bar{V}_\alpha(r)$	pK_{AHY}
g^d_g^-	-550.957677	0.0	2	39.1	0.3409			-549.986058	0.0	2	40.5		
pl^d	-550.957671	0.0	1	19.4	0.3466	0.3423	1.92	-549.985920	0.4	1	17.5	0.3422	1.91
pl^p	-550.957384	0.8	1	14.3	0.3449			-549.985719	0.9	1	14.1		
g^p_g^-	-550.957336	0.9	2	27.2	0.3399			-549.985707	0.9	2	27.9		

2-fluoro-5-nitrobenzyl alcohol. **6b**

Conformer	IEF-PCM/MPWB1K/6-31+G(d,p)							IEF-PCM/MP2/6-311++G(2d,p)					
	G	Δ*G*	deg.	p_i	$V_\alpha(r)$	$\bar{V}_\alpha(r)$	pK_{AHY}	*G*	Δ*G*	deg.	p_i	$\bar{V}_\alpha(r)$	pK_{AHY}
pl^d	-650.182594	0.0	1	36.6	0.3483			-649.096388	0.0	1	26.8		
g^d_g^-	-650.182170	1.1	2	46.7	0.3462			-649.095975	1.1	2	34.6		
g^p_*chel*	-650.180425	5.7	2	7.4	0.3314	0.3453	2.07	-649.095040	3.5	2	12.9	0.3432	1.96
g^p_g^-	-650.180030	6.7	2	4.8	0.3389			-649.095014	3.6	2	12.5		
g^p_*t*	-650.179962	6.9	2	4.5	0.3402			-649.095061	3.5	2	13.2		

2,6-difluoro-3-nitrobenzyl alcohol. **6c**

Conformer	IEF-PCM/MPWB1K/6-31+G(d,p)							IEF-PCM/MP2/6-311++G(2d,p)					
	G	Δ*G*	deg.	p_i	$V_\alpha(r)$	$\bar{V}_\alpha(r)$	pK_{AHY}	*G*	Δ*G*	deg.	p_i	$\bar{V}_\alpha(r)$	pK_{AHY}
$g^{p,s}$_*chel*	-749.392621	0.0	1	23.1	0.3334	0.3365	1.61	-748.193569	1.4	1	17.2	0.3379	1.69
$g^{p,a}$_*chel*	-749.392444	0.5	1	19.2	0.3340			-748.193389	1.9	1	14.2		

$g^{d,a}$_chel	-749.392430	0.5	1	18.9	0.3354			-748.193323	2.0	1	13.2		
$perp^{s}$_t	-749.392418	0.5	1	18.6	0.3417			-748.194101	0.0	1	30.2		
$perp^{a}$_t	-749.391864	2.0	1	10.4	0.3419			-748.193619	1.3	1	18.1		
$g^{d,s}$_chel	-749.391819	2.1	1	9.9	0.3350			-748.192721	3.6	1	7.0		

Phenyl ethanol – 1-α-methylbenzyl alcohol. **7a**

Conformer	IEF-PCM/MPWB1K/6-31+G(d,p)							IEF-PCM/MP2/6-311++G(2d,p)					
	G	ΔG	deg.	p_i	$V_\alpha(r)$	$\bar{V}_\alpha(r)$	pK_{AHY}	G	ΔG	deg.	p_i	$\bar{V}_\alpha(r)$	pK_{AHY}
g_g^{-}	-385.802492	0.0	2	79.4	0.3208			-385.017774	0.0	2	88.7		
g_t	-385.800623	4.9	2	11.0	0.3276	0.3215	0.83	-385.015496	6.0	2	8.0	0.3213	0.82
g^{perp}_g^{-}	-385.800496	5.2	2	9.6	0.3199			-385.014672	8.1	2	3.3		

2-fluorophenyl ethanol-1. **7b**

Conformer	IEF-PCM/MPWB1K/6-31+G(d,p)							IEF-PCM/MP2/6-311++G(2d,p)					
	G	ΔG	deg.	p_i	$V_\alpha(r)$	$\bar{V}_\alpha(r)$	pK_{AHY}	G	ΔG	deg.	p_i	$\bar{V}_\alpha(r)$	pK_{AHY}
$g^{d,perp}$_t	-485.025894	0.0	2	34.0	0.3308			-484.125871	0.0	2	25.4		
$g^{d,perp}$_g^{-}	-485.025553	0.9	2	23.7	0.3298			-484.125534	0.9	2	17.8		
$g^{p,perp}$_chel	-485.025343	1.5	2	19.0	0.3090			-484.125769	0.3	2	22.8		
$g^{p,perp}$_g^{-}	-485.024662	3.2	2	9.2	0.3212	0.3232	0.92	-484.125614	0.7	2	19.3	0.3218	0.84
g^{p}_chel	-485.024522	3.6	2	7.9	0.3090			-484.124479	3.7	2	5.8		
g^{p}_g^{-}	-485.023537	6.2	2	2.8	0.3203			-484.123811	5.4	2	2.9		
$g^{p,perp}$_t	-485.023217	7.0	2	2.0	0.3232			-484.124215	4.4	2	4.4		
g^{p}_t	-485.022884	7.9	2	1.4	0.3233			-484.123254	6.9	2	1.6		

2,5-difluoro-phenyl ethanol-1. **7c**

Conformer	IEF-PCM/MPWB1K/6-31+G(d,p)							IEF-PCM/MP2/6-311++G(2d,p)					
	G	ΔG	deg.	p_i	$V_\alpha(r)$	$\bar{V}_\alpha(r)$	pK_{AHY}	G	ΔG	deg.	p_i	$\bar{V}_\alpha(r)$	pK_{AHY}
$g^{p,perp}$_chel	-584.249066	0.0	4	55.3	0.3134			-583.234731	0.0	4	47.4		
$g^{p,perp}$_g^{-}	-584.352073	1.1	4	35.3	0.3246	0.3185	0.67	-583.234593	0.4	4	40.9	0.3194	0.72
$g^{p,perp}$_t	-584.247354	4.5	4	9.0	0.3260			-583.233383	3.5	4	11.4		
g^{p}_chel	-584.244375	12.3	4	0.4	0.3144			-583.229856	12.8	4	0.3		

2-methyl benzyl alcohol. **8a**

Conformer	IEF-PCM/MPWB1K/6-31+G(d,p)							IEF-PCM/MP2/6-311++G(2d,p)					
	G	ΔG	deg.	p_i	$V_\alpha(r)$	$\bar{V}_\alpha(r)$	pK_{AHY}	G	ΔG	Deg.	p_i	$\bar{V}_\alpha(r)$	pK_{AHY}
pl	-385.799729	0.0	1	32.8	0.3311			-385.014164	2.2	1	14.0		
g^{p}_g^{-}	-385.799251	1.3	2	39.6	0.3200	0.3253	1.03	-385.015001	0.0	2	68.2	0.3226	0.89
g^{d}_g^{-}	-385.798755	2.6	2	23.4	0.3260			-385.013206	4.7	2	10.2		
g^{p}_t	-385.797131	6.8	2	4.2	0.3258			-385.012923	5.5	2	7.5		

2-fluoro-5-methylbenzyl alcohol. **8b**

Conformer	IEF-PCM/MPWB1K/6-31+G(d,p)							IEF-PCM/MP2/6-311++G(2d,p)					
	G	ΔG	deg.	p_i	$V_\alpha(r)$	$\bar{V}_\alpha(r)$	pK_{AHY}	G	ΔG	Deg.	p_i	$\bar{V}_\alpha(r)$	pK_{AHY}
$g^p\_g^-$	-485.025555	0.0	2	78.5	0.3227	0.3218	0.84	-484.126377	0.0	2	79.9	0.3220	0.85
g^p_chel	-485.023731	4.8	2	11.4	0.3102			-484.124409	5.2	2	9.9		
g^d_t	-485.023626	5.1	2	10.2	0.3280			-484.124428	5.1	2	10.1		

2-t-butylbenzyl alcohol. **9a**

Conformer	IEF-PCM/MPWB1K/6-31+G(d,p)							IEF-PCM/MP2/6-311++G(2d,p)					
	G	ΔG	deg.	p_i	$V_\alpha(r)$	$\bar{V}_\alpha(r)$	pK_{AHY}	G	ΔG	Deg.	p_i	$\bar{V}_\alpha(r)$	pK_{AHY}
$g^d\_g^-$	-503.590121	0.0	2	42.4	0.3180	0.3184	0.67	-502.545573	0.3	2	40.8	0.3177	0.63
$g^{p,d}\_g^-$	-503.590059	0.2	2	39.7	0.3152			-502.545685	0.0	2	45.9		
$g^{d,s}\_t$	-503.588456	4.4	2	7.3	0.3292			-502.542576	8.2	2	1.7		
$perp^d\_t$	-503.588312	4.8	2	6.2	0.3247			-502.544077	4.2	2	8.4		
$g^s\_g^-$	-503.587799	6.1	2	3.6	0.3226			-502.542953	7.2	2	2.5		
$g^{p,s}\_t$	-503.586410	9.7	2	0.8	0.3283			-502.541822	10.1	2	0.8		

2-fluoro-5-t-butylbenzyl alcohol. **9b**

Conformer	IEF-PCM/MPWB1K/6-31+G(d,p)							IEF-PCM/MP2/6-311++G(2d,p)					
	G	ΔG	deg.	p_i	$V_\alpha(r)$	$\bar{V}_\alpha(r)$	pK_{AHY}	G	ΔG	Deg.	p_i	$\bar{V}_\alpha(r)$	pK_{AHY}
$g^{p,d}\_chel$	-602.816405	0.0	2	86.5	0.3112	0.3130	0.39	-601.656678	0.0	2	82.6	0.3135	0.41
$g^{p,d}\_t$	-602.814447	5.1	2	10.9	0.3244			-601.655126	4.1	2	15.9		
$g^{p,s}\_g^-$	-602.812936	9.1	2	2.2	0.3255			-601.652685	10.5	2	1.2		
$g^{d,s}\_t$	-602.811424	13.1	2	0.4	0.3297			-601.651369	13.9	2	0.3		

3 Detailed results from AIM analysis

Table S2. Optimized distances d(H···F) (Å) and densities (ρ) and laplacian ($\nabla^2\rho$) of AIM critical point obtained at IEFPCM(radii=UFF)/MPWB1K/6-31+G(d,p) level of theory; Population (%) of conformers which possess intramolecular H···F interaction, and relative free energies ΔG (kJ·mol^{-1}) obtained after single point MP2 correction

Compound	Conformer	% conf	ΔG (kJ mol^{-1})	d(H···F) Å	density AIM, ρ	$\nabla^2\rho(r)$	total energy, H, *10^{-3}	E_{HB} (kJ mol^{-1})
**1b**	g^p_chel	21	1.80	2.252	0.0125	0.0563	1.107	15.6
**2b**	$g^{p,Z}\_chel$	16	1.28	2.224	0.0130	0.0577	0.984	16.4
	$g^{p,E}\_chel$	8	2.83	2.230	0.0132	0.0583	0.960	16.6
**3b**	g^p_chel	17	3.50	2.268	0.0121	0.0556	1.200	15.1
**4b**	$g^{p,Z}\_chel$	1	8.74	2.252	0.0125	0.0568	1.134	15.7
	$g^{p,E}\_chel$	2	5.68	2.317	0.0132	0.0586	1.023	16.6

	$g^{p,s}$_chel	10	1.99	2.291	0.0128	0.0576	1.123	16.0
	$g^{p,a}$_chel	9	2.24	2.297	0.0116	0.0541	1.309	14.3
**5b**	$g^{p,a}$_chel	2	6.38	2.259	0.0124	0.0568	1.188	15.5
	$g^{p,s}$_chel	2	6.64	2.255	0.0125	0.0571	1.171	15.7
**6b**	g^{p}_chel	13	3.54	2.287	0.0119	0.0555	1.340	14.7
**7b**	$g^{p,perp}$_chel	23	0.27	2.187	0.0144	0.0616	0.725	18.3
	g^{p}_chel	6	3.65	2.166	0.0149	0.0633	0.629	19.1
**8b**	g^{p}_chel	10	5.17	2.223	0.0134	0.0593	0.987	16.9
**9b**	g^{p}_chel	83	0.00	2.251	0.0129	0.0592	1.215	16.2
**1c**	*g_chel*	70	0.00	2.252	0.0125	0.0567	1.131	15.7
**2c**	$g^{p,E}$_chel	29	0.16	2.255	0.0124	0.0564	1.139	15.5
	$g^{d,E}$_chel	15	1.83	2.227	0.0131	0.0583	0.993	16.5
	$g^{p,Z}$_chel	6	3.84	2.224	0.0134	0.0595	1.015	16.9
	$g^{d,Z}$_chel	10	2.90	2.315	0.0110	0.0520	1.379	13.5
**3c**	g^{p}_chel	33	0.42	2.330	0.0106	0.0512	1.449	13.0
	g^{d}_chel	29	0.75	2.239	0.0129	0.0579	1.081	16.2
**4c**	$g^{p,Z}$_chel	3	5.73	2.349	0.0103	0.0504	1.512	12.6
	$g^{d,Z}$_chel	1	7.91	2.246	0.0127	0.0575	1.121	15.9
	$g^{p,E}$_chel	0	10.62	2.302	0.0113	0.0534	1.370	13.9
	$g^{d,E}$_chel	0	11.22	2.242	0.0129	0.0581	1.112	16.1
	$g^{p,s}$_chel	27	0.00	2.289	0.0117	0.0545	1.322	14.4
	$g^{d,s}$_chel	10	2.52	2.300	0.0114	0.0536	1.357	14.0
	$g^{p,a}$_chel	10	2.31	2.335	0.0107	0.0514	1.476	13.0
	$g^{d,a}$_chel	14	1.57	2.300	0.0114	0.0537	1.358	14.1
**5c**	g^{p}_chel	14	2.94	2.356	0.0103	0.0506	1.558	12.5
	g^{d}_chel	12	3.40	2.272	0.0121	0.0559	1.260	15.0
**6c**	$g^{p,s}$_chel	17	1.40	2.280	0.0111	0.0540	1.555	13.6
	$g^{d,s}$_chel	7	3.62	2.266	0.0121	0.0565	1.320	15.1
	$g^{p,a}$_chel	14	1.87	2.309	0.0112	0.0544	1.532	13.8
	$g^{d,a}$_chel	13	2.04	2.275	0.0116	0.0549	1.404	14.4
**7c**	$g^{p,perp}$_chel	47	0.00	2.179	0.0148	0.0624	0.856	18.2
	g^{p}_chel	0	12.8	2.194	0.0143	0.0635	0.737	18.9

4 Detailed results of NBO analysis

Table S3. NBO Analysis (kJ mol^{-1})

Compound	Conformer	n_F - σ^*_{OH}	n_O - $\sigma^*_{C1-C\alpha}$	σ_{C1-C7} - σ^*_{OH}	σ_{OH} - $\sigma^*_{C1-C\alpha}$	n_O - $\sigma^*_{=CH}$	$\pi_{C1=C6}$ - σ^*_{CO}	σ_{CO} - $\pi^*_{C1=C6}$	σ_{CO} - $\sigma^*_{C1=C2}$	$\sigma_{C1=C2}$ - $\sigma^*_{C\alpha-O}$	n_{F2} - $\sigma^*_{C\alpha-O}$	n_O - $\pi^*_{C1=C6}$	n_O - $\sigma^*_{C=C}$
**1a**	g_g^-		30.3				9.0		7.1	4.6			
	pl		7.3	8.4	13.0	3.6			9.9	5.3			
**1b**	$g^d\_g^-$		36.9								2.1		
	g^p_chel	4.6	37.1				26.5						
	$perp\_t$		5.9	8.5	11.8		33.0	8.7					
	$g^p\_g^-$		31.5				31.8	7.0	3.3			6.0	
	pl^d		7.8	8.8	12.4	3.5				4.8	2.7		
**1c**	g_chel	4.4	38.6				24.8	5.6					
	$perp\_t$		7.1	9.1	11.8		32.0	8.9					
**3a**	$g^d\_g^-$		33.1				6.5		7.2/6.3	5.0			
	$g^p\_g^-$		33.5						7.4	4.9			
	pl^d		7.3	8.5	13.1	3.3			9.6	5.5			
	pl^p		7.5	8.4	13.3	3.6			9.9	5.3			
**3b**	$g^p\_g^-$		33.9				29.1	7.3				6.5	2.5
	$g^d\_g^-$		39.4						7.4	5.1	2.1		
	g^p_chel	4.3	38.5				24.8	5.7	4.8				5.0
	$perp\_t$		6.2	8.7	11.9		30.9	9.2					
	pl^d		8.1	8.8	12.7	3.3				4.8	2.7		
**3c**	g^d_chel	4.7 [a]	39.5				22.8	5.7	4.1			5.0	
	g^p_chel	3.2	40.2				22.0	5.2	4.1			3.1	
	$perp\_t$		7.3	9.3	11.9		30.1	9.5					
**6a**	$g^d\_g^-$		36.1				9.7		7.4	4.5			
	$g^p\_g^-$		36.8						8.0/4.6	4.9			2.1
	pl^d		7.7	8.4	13.2	3.8			10.0	5.1			
	pl^p		7.8	8.6	13.2	3.9			10.4	4.8			
**6b**	$g^p\_g^-$		36.7				29.9	6.9	3.5			6.5	
	$g^d\_g^-$		42.3			2.3			8.0	4.8	2.5		2.8
	g^p_chel	3.8	40.4				25.6	5.5	5.0				
	g^p_t		5.9	8.7	11.6		29.9	8.4	3.7				
	pl^d		8.4	9.0	12.6	3.8			9.7	4.3	2.6		
**6c**	$g^{p,a}\_chel$	3.1	42.2						4.0				
	$g^{d,s}\_chel$	3.3 [a]	41.9				24.5	5.7	4.1			5.4	
	$g^{d,a}\_chel$	3.8 [a]	41.7				24.1	5.8	4.2			5.4	
	$g^{p,s}\_chel$	2.9	42.1				24.0	5.1	4.1			3.2	
	$perp^s\_t$		7.5	9.4	11.9		31.2	8.9					
	$perp^a\_t$		7.6	9.4	11.9		31.2	9.1					

5 Experimental procedures

5.1 2-Fluorobenzyl alcohol

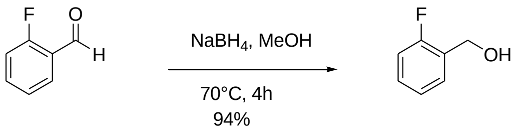

2-Fluorobenzaldehyde (2.55 mL, 24.17 mmol) was dissolved in THF (25 mL) and sodium borohydride (0.914, 24.17 mmol) was added to the reaction mixture. MeOH (5 mL) was then added drop wise and the mixture was stirred at 70 °C for 4h. After total consumption of the starting material, the reaction mixture was washed with water (2×10 mL) and brine (2×10 mL). The obtained organic layer was dried ($MgSO_4$), filtered, concentrated under reduced pressure and purified by column chromatography using ethyl acetate / petroleum ether (20/80). This afforded 2.87 g (94%) of the corresponding benzyl alcohol as an oil. **^{1}H NMR** (300 MHz, $CDCl_3$, ppm) δ 7.43 (1H, td, *J*=7.5, *J*=1.6 Hz, H_{Ar}), 7.24–7.34 (1H, m, H_{Ar}), 7.15 (1H, td, *J*=7.4, 0.9 Hz, H_{Ar}), 7.01–7.10 (1H, m, H_{Ar}), 4.77 (2H, d, *J*=5.8 Hz, CH_2), 1.92 (1H, t, *J*=6.0 Hz, OH); **^{13}C NMR** (75 MHz, $CDCl_3$, ppm) δ 160.6 (C^{IV}, d, *J*=247 Hz, C_2), 129.3 (CH, s, C_4 or C_6), 129.3 (CH, d, *J*=13 Hz, C_4 or C_6), 127.8 (C^{IV}, d, *J*=15 Hz, C_1), 124.2 (CH, d, *J*=4 Hz, C_5), 115.2 (CH, d, *J*=21 Hz, C_3), 59.4 (CH_2, d, *J*=4 Hz); **^{19}F NMR** (282 MHz, $CDCl_3$, ppm) δ -120.1 (1F, br. s.).

Already described by Nadim S. Shaikh, Kathrin Junge, and Matthias Beller, in *Org. Lett.*, **2007**, *9*, 26, 5429–5432

5.2 2-Fluoro-5-methoxylbenzyl alcohol

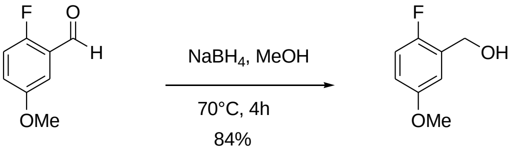

The 2-Fluoro-5-nitrobenzyl alcohol was obtained using the same procedure described for the 2-Fluorobenzyl alcohol starting from 3.0 g of starting material (19.46 mmol) and afforded 2.57 g (84%) of the corresponding benzyl alcohol as a colourless liquid. **^{1}H NMR** (300 MHz, $CDCl_3$, ppm) δ 6.92–7.01 (2H, m, H_{Ar}), 6.77 (1H, dt, *J*=8.8 Hz, *J*=3.7 Hz, H_{Ar}), 4.73 (2H, br. d, *J*=5.5 Hz, CH_2), 3.79 (3H, s, OMe), 2.00 (1H, t, *J*=5.9 Hz, OH); **^{13}C NMR** (75 MHz, $CDCl_3$, ppm) δ 155.8 (C^{IV}, d, *J*=2 Hz, C_5), 154.8 (C^{IV}, d, *J*=238 Hz, C_2), 128.4 (C^{IV}, d, *J*=17 Hz, C_1), 115.7 (CH, d, *J*=23 Hz, C_3), 114.1 (CH, d, *J*=8 Hz, C_4 or C_6), 113.8 (CH, d, *J*=5 Hz, C_4 or C_6), 59.4 (CH_2, d, *J*=4 Hz), 55.7 (OCH_3, s); **^{19}F NMR** (282 MHz, $CDCl_3$, ppm) δ -130.9 (1F, br. s.).

Already described by Jaeyoung Lee, Taekyu Ryu, SangJune Park, and Phil Ho Lee, in *J. Org. Chem.* **2012**, 77, 4821−4825

5.3 2-Fluoro-5-nitrobenzyl alcohol

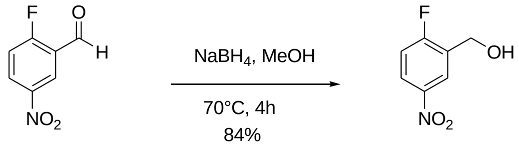

The 2-Fluoro-5-nitrobenzyl alcohol was obtained using the same procedure described for the 2-Fluorobenzyl alcohol starting from 3.0 g of starting material (17.74 mmol) and afforded 2.55 g (84%) of the corresponding benzyl alcohol as a pale yellow solid; **^{1}H NMR** (300 MHz, $CDCl_3$, ppm) δ 8.42 (1H, dd, *J*= 6.1, *J*=2.8 Hz, H_6), 8.19 (1H, ddd, *J*=8.8, *J*=4.2, *J*=3.2 Hz, H_4), 7.19 (1H, t, *J*=8.9 Hz, H_3), 4.85 (2H, d, *J*=5.9 Hz, CH_2), 2.24 (1H, t, *J*=5.9 Hz, OH); **^{13}C NMR** (100 MHz, $CDCl_3$, ppm) δ 163.5 (C^{IV}, d, *J*=257 Hz, C_1), 144.1 (C^{IV}, s, C_5), 129.8 (C^{IV}, d, *J*=17 Hz, C_1), 125.0 (CH, d, *J*=10 Hz, C_4 or C_6), 125 (CH, d, *J*=7 Hz, C_4 or C_6), 116.1 (CH, d, *J*=24 Hz, C_3), 58.3 (CH_2, d, *J*=4 Hz); **^{19}F NMR** (282 MHz, $CDCl_3$, ppm) δ -108.7 (1F, br. s.); **HRMS** (EI pos.) m/z 171.03367 $[M]^+$ (Calcd. 171.03317).

Already described by A. Richard Bunce, David Rogers, Takahiro Nago, Scott A. Bryant, in *J. Heterocyclic Chem.* **2008**, 45, 2, 547-550

5.4 2,6-difluoro-3-nitrobenzyl alcohol

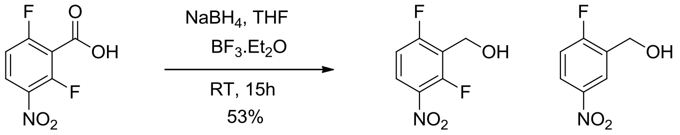

To a suspension of $NaBH_4$ (1.9eq, 25.1mmol) in 44 mL of THF at 0 °C was added the carboxylic acid (13.2mmol) dissolved in 12mL of THF over a period of 30 minutes. Then, 4.45mL of boronetrifluoride etherate were added dropwise over a period of 30 minutes. The mixture was stirred at room temperature for 15 hours and was then quench by adding dropwise HCl 1N until no gas comes out anymore. The solution is diluted with water and extract 3 times with ethyl acetate. The organic layer is dried and the solvent was evaporated. The crude mixture was purified by flash chromatography using the petrol ether / ethyl acetate (86/14 to 82/18) as eluent and the fractions were purified by HPLC (Hexane/Ethyl acetate: 78/22) which permitted to afford 1.23 g of the 2,6-difluoro-3-nitrobenzyl alcohol as a white solid (53%) and the 2-fluoro-5-nitrobenzyl alcohol (35%).

^{1}H NMR (400MHz, $CDCl_3$, ppm) δ 8.12 (CH, ddd, *J*=14.5Hz, *J*=8.8 Hz, *J*=5.7Hz, H_4), 7.08 (CH, ddd, *J*=9.4Hz, *J*=8.2Hz, *J*=1.7Hz, H_5), 4.86 (CH_2, d, *J*=6.5Hz), 2.09 (OH, t, *J*=6.6Hz); **^{13}C NMR** (100MHz, $CDCl_3$, ppm) δ 164.2 (CF, dd, *J*=260Hz, *J*=7Hz, C_2), 155.3 (CF, dd, *J*=267Hz, *J*=9Hz, C_6), 134.3 (C^{IV}, m, C_3), 127.1 (CH_{ar}, dd, *J*=12Hz, *J*=2Hz, C_4), 119.0 (C^{IV}, dd, *J*=21Hz, *J*=18Hz, C_1), 112.0 (CH_{ar}, dd, *J*=24Hz, *J*=4Hz, C_5), 52.7 (CH_2, dd, *J*=4Hz, *J*=4Hz); **^{19}F NMR** (376MHz, $CDCl_3$, ppm) δ -103.45 (dt, *J*=13.6Hz, *J*=6.9Hz), -117.83 (dd, *J*=12.6Hz, *J*=9.1Hz); **MS (EI):** m/z 189 $[M]^+$ (100%); **HRMS** (EI pos.): m/z 189.02375 $[M]^+$ (Calcd. 189.02322); **IR** (cm^{-1}, dissolved in chloroform) 3343.9 (m, br), 1623.6 (s), 1596.9 (s), 1530.1 (s), 1475.7 (s), 1348.8 (s), 1276.9 (s), 1220.4 (s), 1056.3 (s), 1020.2 (s), 966.2 (m), 831.1 (m), 767.9 (m), 733.5 (m), 696.2 (m), 617.3 (m); **mp** 54-56 °C.

Already described by Yoshiki Yoshida, David Barrett, Hidenori Azami, Chizu Morinaga, Satoru Matsumoto, Yoshimi Matsumoto and Hisashi Takasugi, in *Bioorg. Med. Chem.* **1999**, 7, 2647-2666

The unexpected selective monodefluorination was observed when the reduction reaction was also carried out starting with the corresponding ester using the sodium boron hydride or lithium aluminium hydride. The procedures are described below.

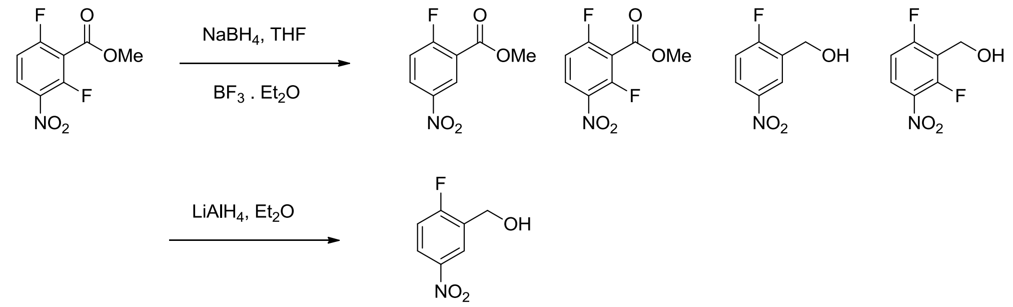

Procedure using $NaBH_4$:

An ice-cooled suspension of sodium borohydride (106mg, 2.81mmol) in 5mL of freshly distilled THF was treated by the methyl 2,6-difluoro-3-nitrobenzoate (1.48mmol, 300mg) over a period of 35 minutes. Then, boron trifluoride etherate (ca. 48% BF_3, 106mg, 4.00mmol) was added over a period of 30 minutes. The mixture was stirred at room temperature for 16 hours and was then quenched by adding dropewise HCl (1N). The resulting solution was diluted with water and extracted 3 times with ethyl acetate. The organic layer was dried over $MgSO_4$ and the solvent was evaporated under reduced pressure. The crude mixture was purified by flash chromatography using petroleum ether / ethyl acetate (96/4 to 78/22) as eluent which permitted to afford 165mg of the mixture of the methyl 2-fluoro-5-nitrobenzoate and the methyl 2,6-difluoro-3-nitrobenzoate (^{19}F NMR ratio: 67/33), followed by 53mg of the 2-fluoro-5-nitrobenzyl alcohol (21%) and 55mg of the 2,6-difluoro-3-nitrobenzyl alcohol (23%).

Procedure using $LiAlH_4$:

To a solution of methyl 2,6-difluoro-3-nitrobenzoate (2.15g, 10.6mmol) in freshly distilled diethyl ether (100mL) was added $LiAlH_4$ (2eq., 21.2mmol, 805mg) over a period of 10 minutes at room temperature. The mixture was stirred overnight, cooled at 0°C and then quench by adding 2.2 mL of water over a period of 1 hour. The solid was filtered on celite and the solvent was evaporated under reduced pressure. The crude mixture was purified by flash chromatography using petroleum ether/ethyl acetate: 86/14 as eluent which permitted to afford the 2-fluoro-5-nitrobenzyl alcohol (35%).

6 Spectra of the compounds

6.1 2-Fluorobenzyl alcohol

au2112lam1.010.esp

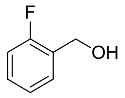

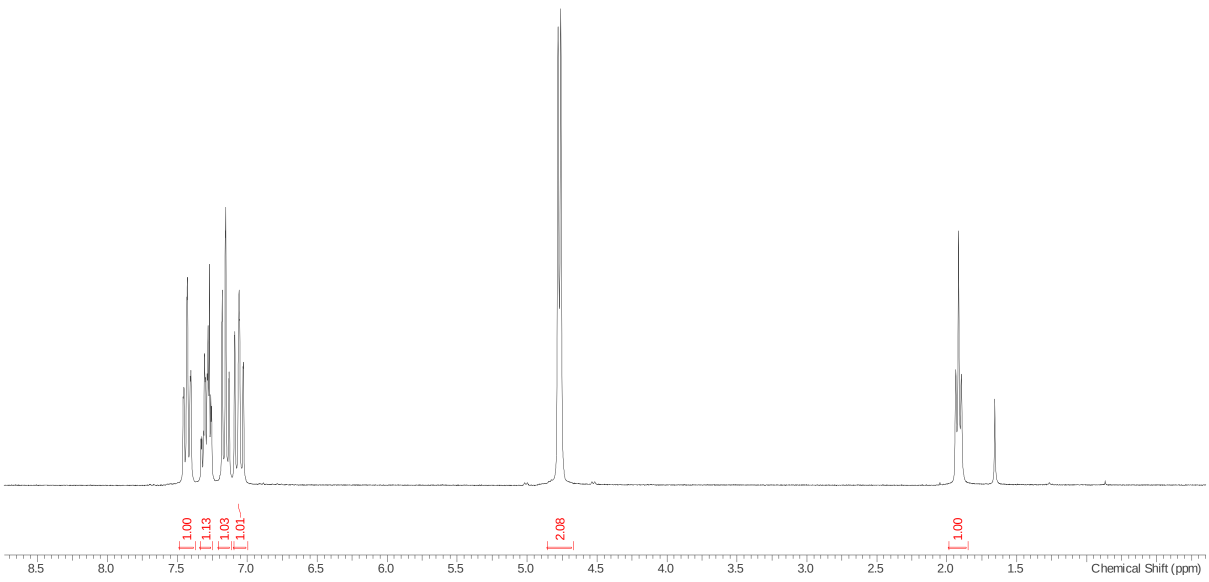

au2112lam1.011.esp

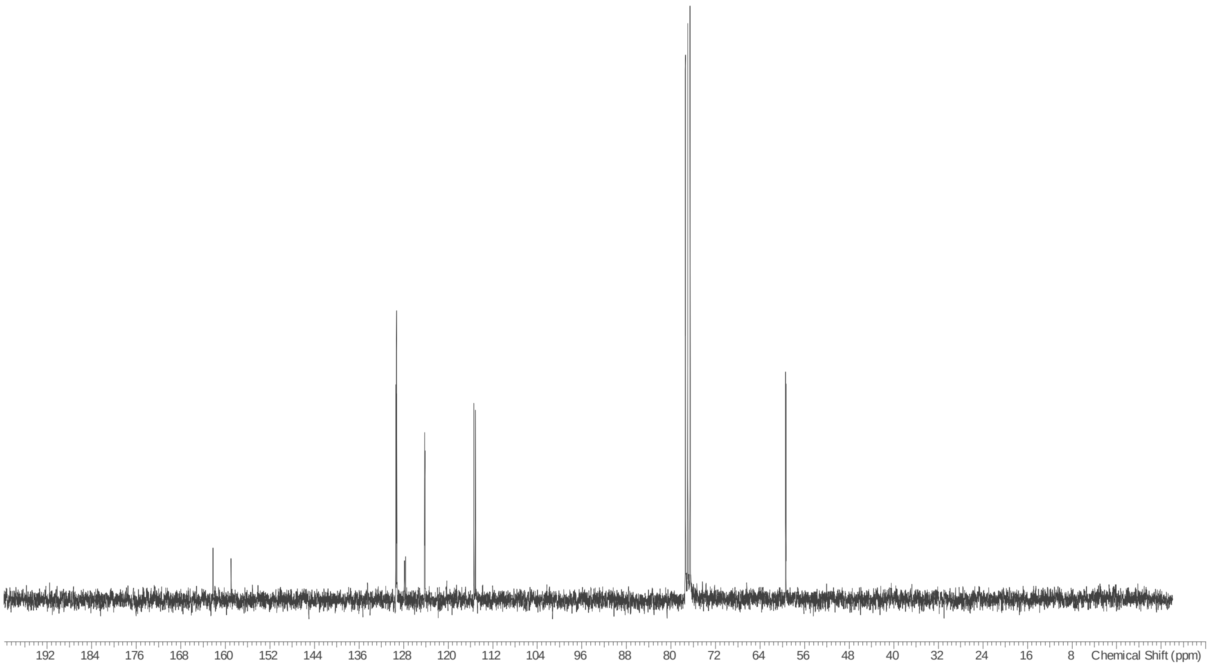

au2112lam1.012.esp

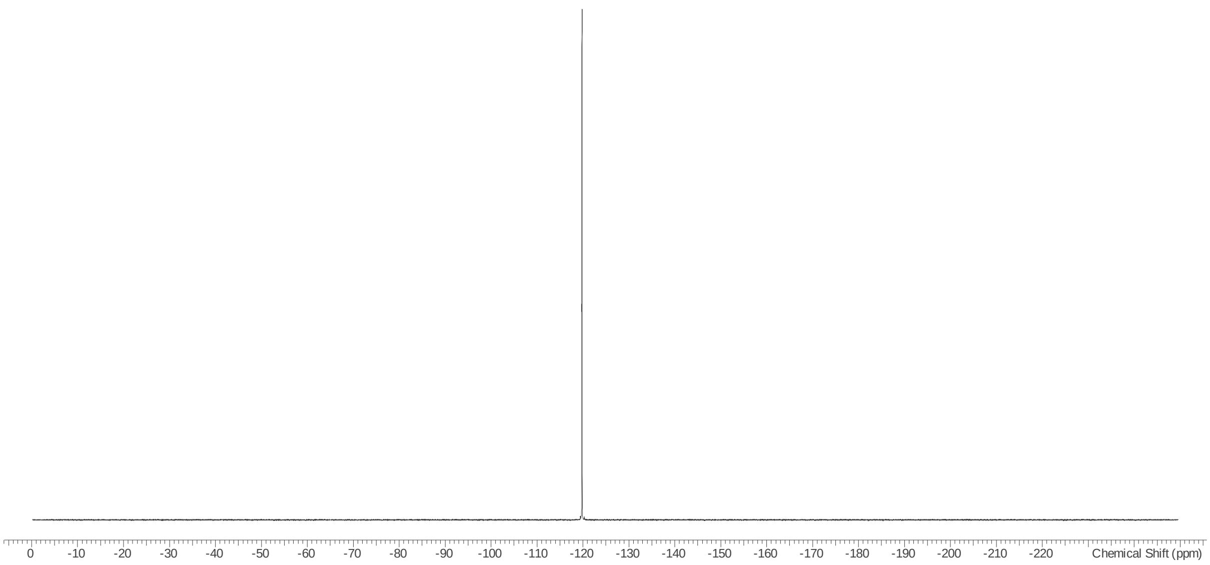

6.2 2-Fluoro-5-methoxybenzyl alcohol

se1112lam1.010.esp

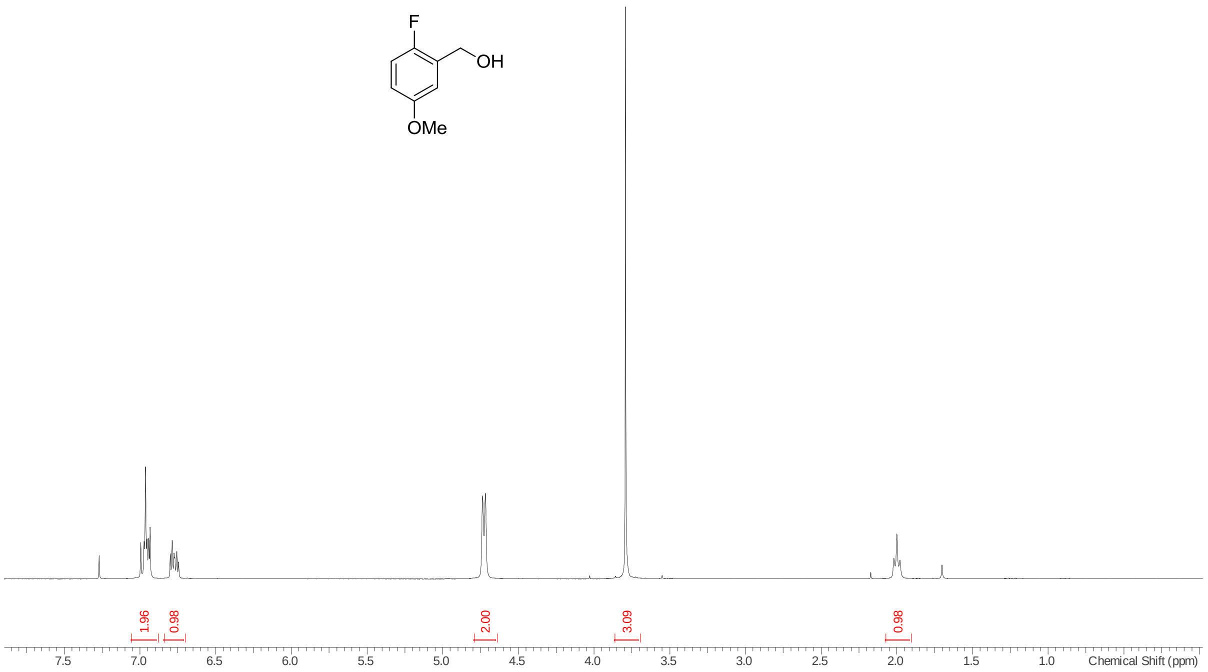

se1112lam1.011.esp

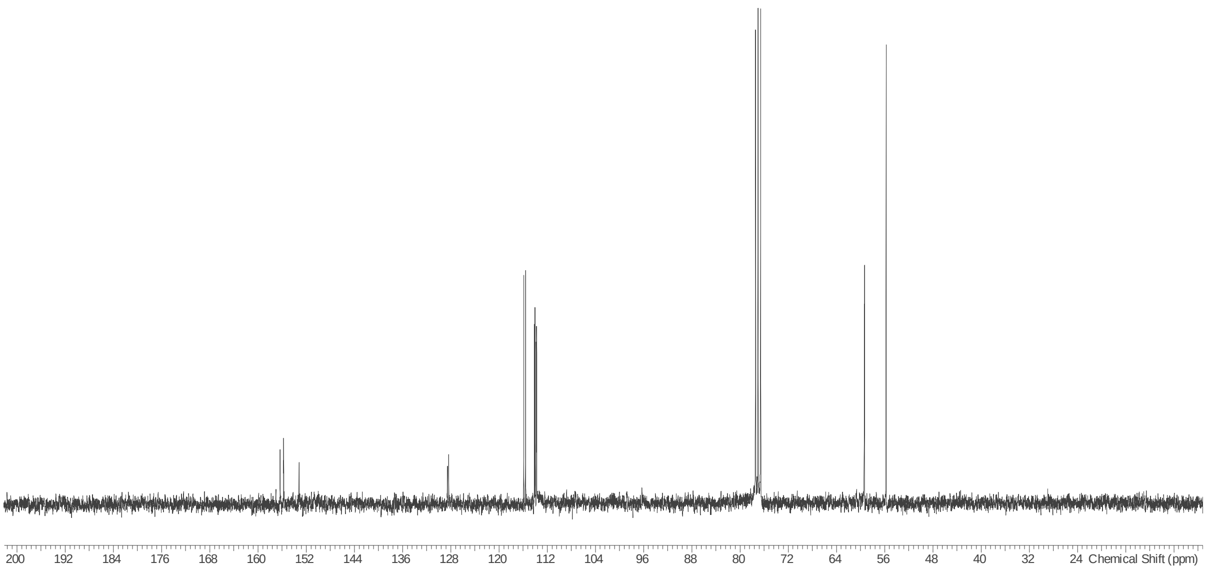

se1112lam1.012.esp

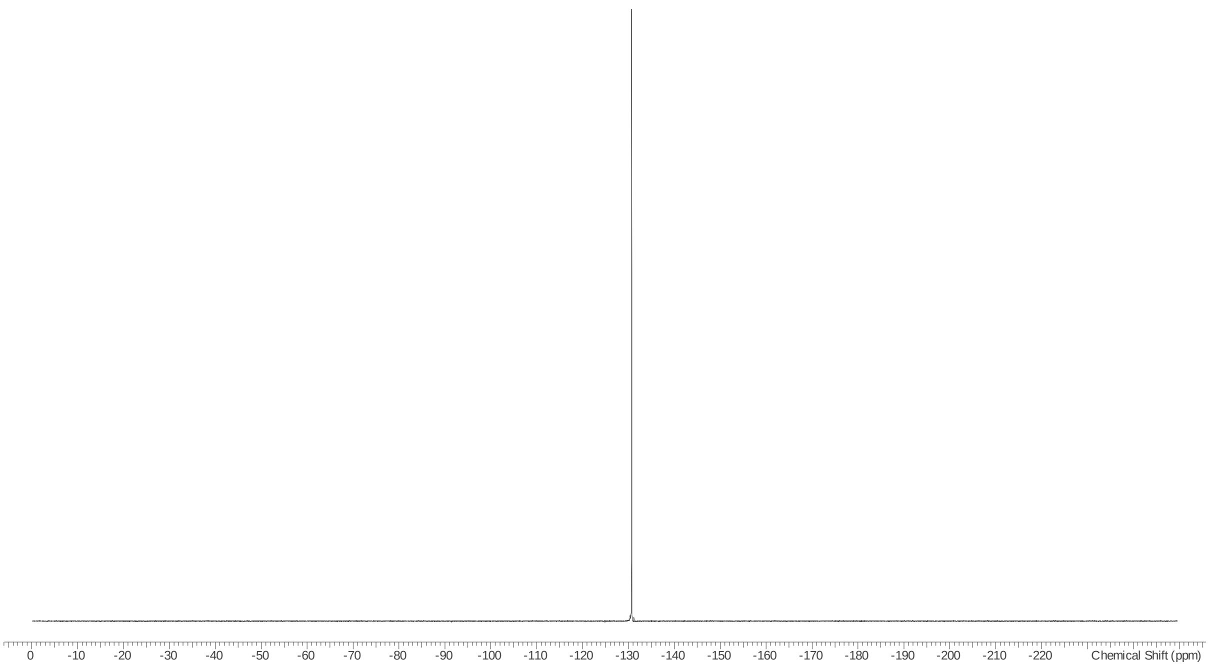

6.3 2-Fluoro-5-nitrobenzyl alcohol

se1812lam1.010.esp

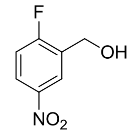

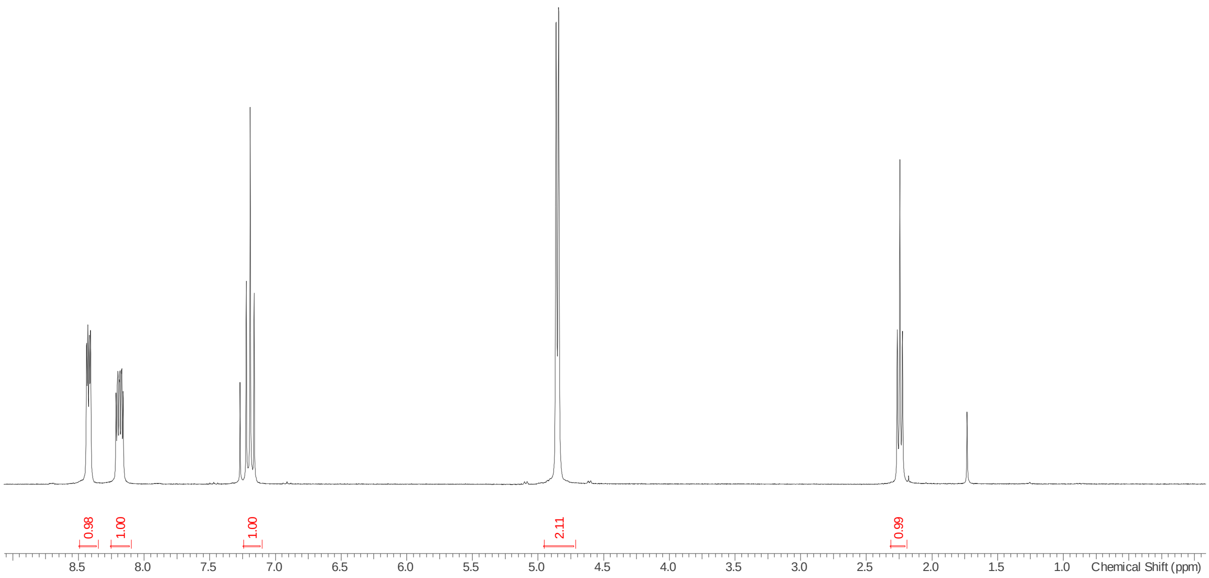

se0214gc8.011.001.1r.esp

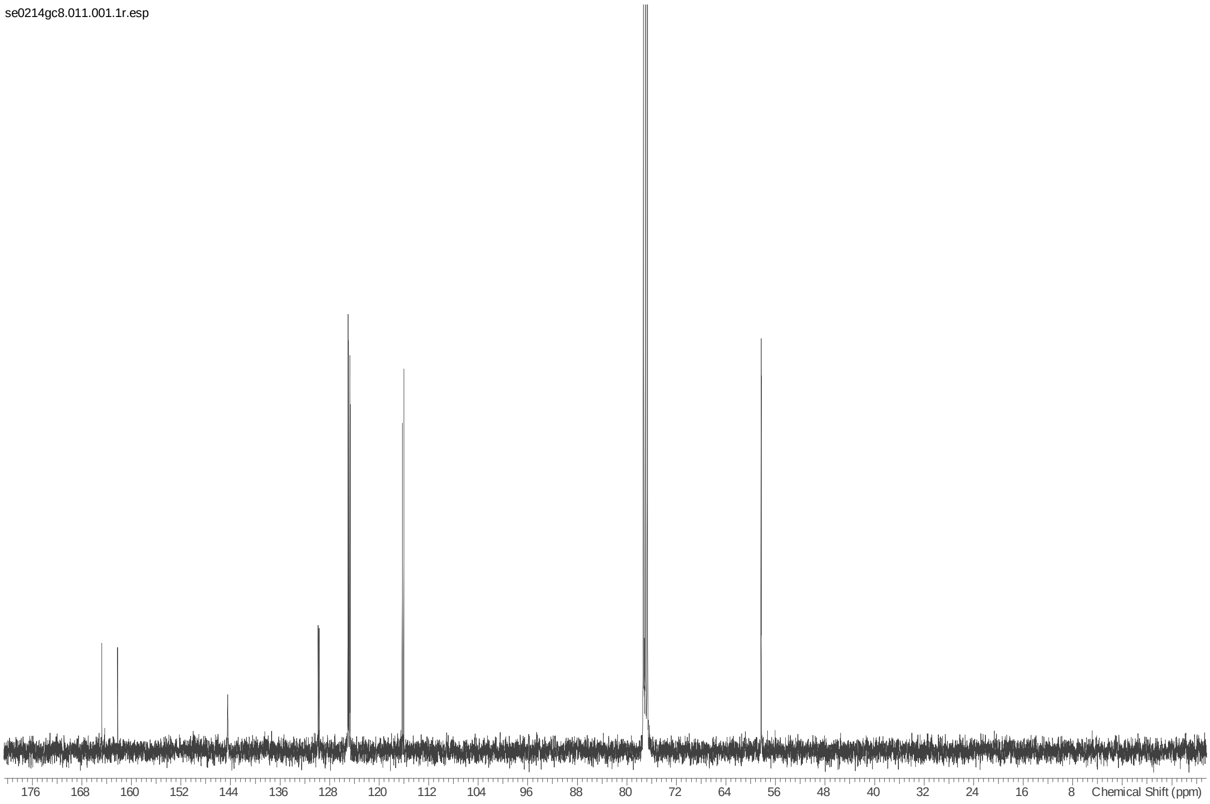

se1812lam1.012.esp

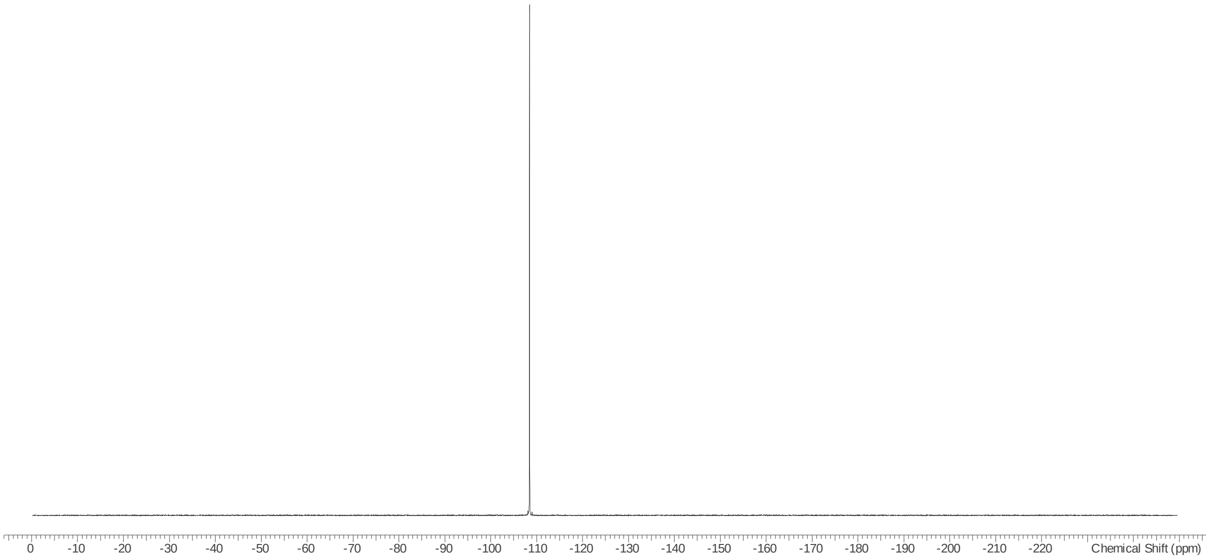

6.4 2,6-difluoro-3-nitrobenzyl alcohol

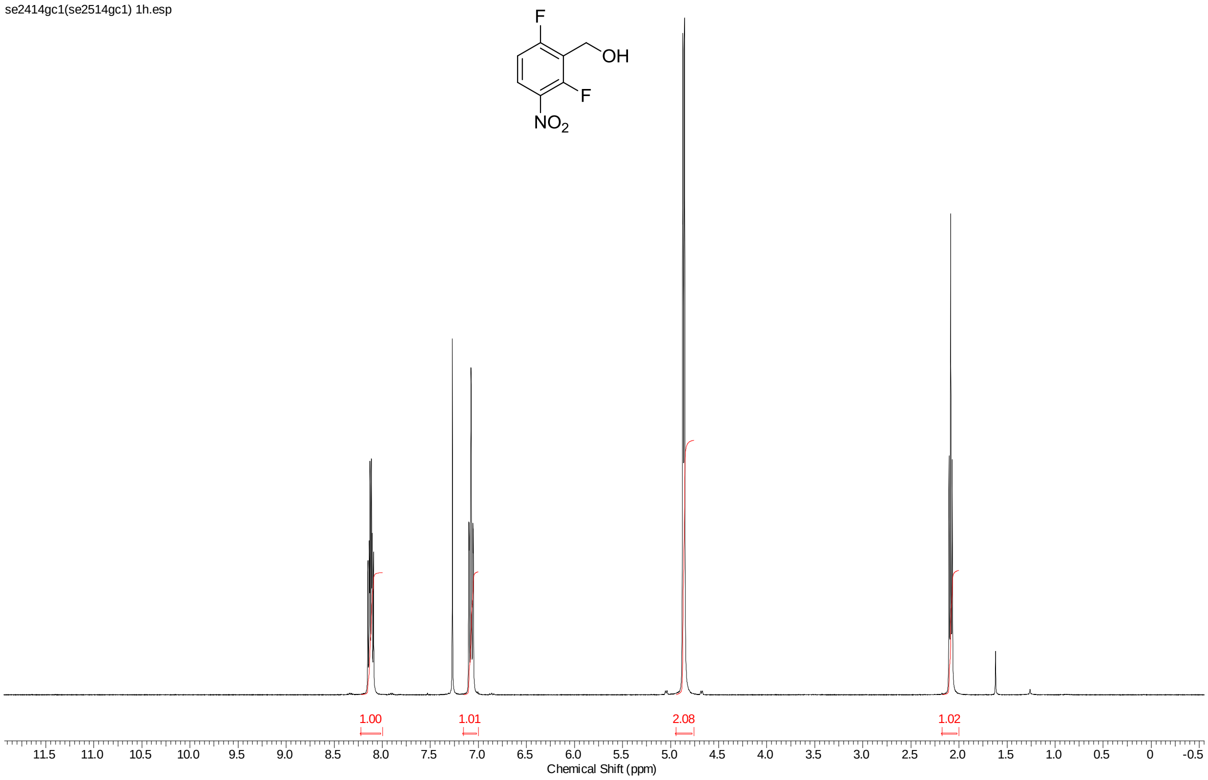

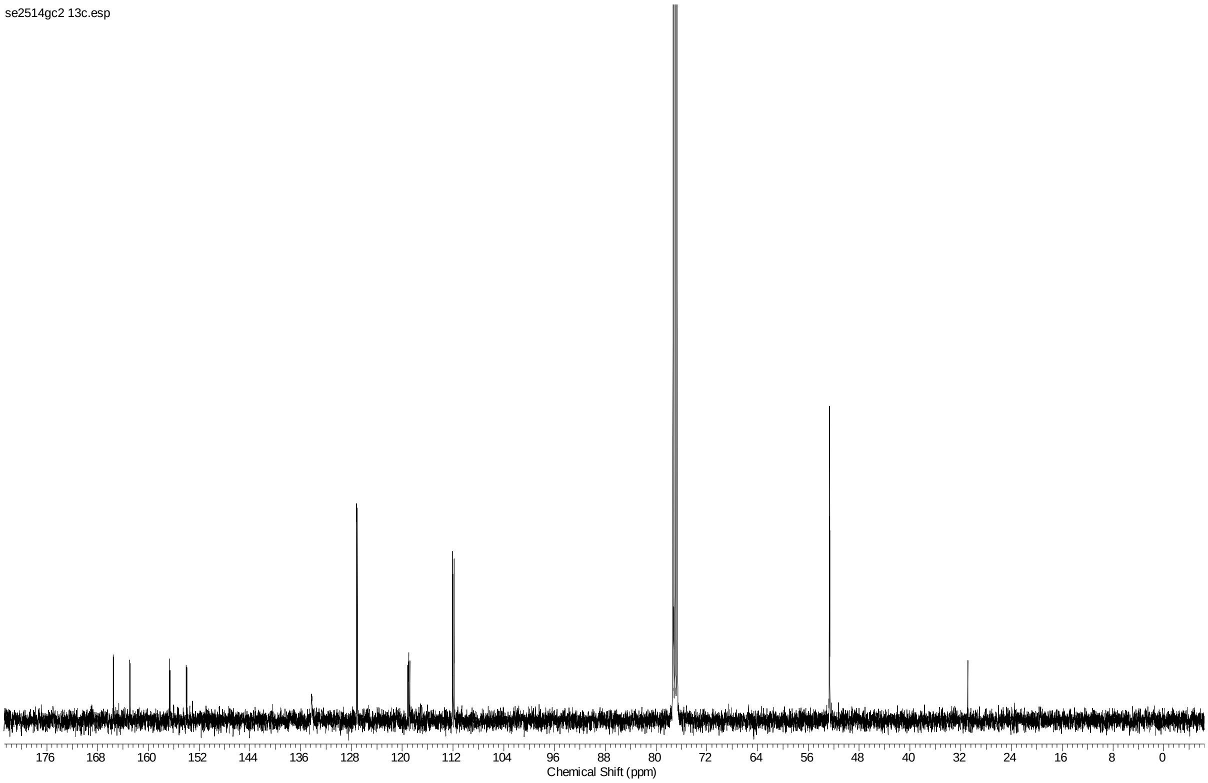

se2514gc2.011.001.1r.esp

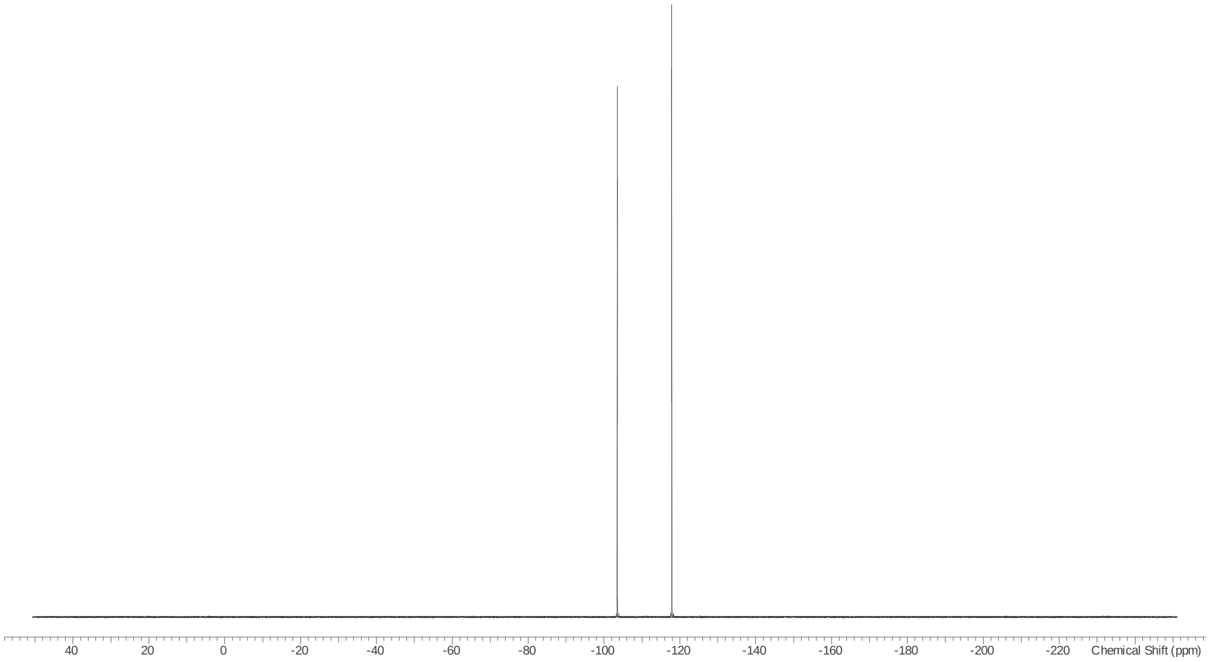

Supplement: Supplementary file 1 — miscellaneous_information [file chem0021-11462-sd1.pdf]
